# Transformation of the recalcitrant pesticide chlordecone by *Desulfovibrio* sp.86 with a switch from ring-opening dechlorination to reductive sulfidation activity

**DOI:** 10.1038/s41598-020-70124-9

**Published:** 2020-08-11

**Authors:** Oriane Della-Negra, Sébastien Chaussonnerie, Nuria Fonknechten, Agnès Barbance, Delphine Muselet, Déborah E. Martin, Stéphanie Fouteau, Cécile Fischer, Pierre-Loïc Saaidi, Denis Le Paslier

**Affiliations:** Génomique Métabolique, Genoscope, Institut François Jacob, CEA, CNRS, Univ Evry, Université Paris-Saclay, 91057 Evry, France

**Keywords:** Microbiology, Bacteria, Pollution remediation, Mass spectrometry, NMR spectroscopy, Environmental monitoring, Structure elucidation, Chemical synthesis

## Abstract

The insecticide chlordecone has been used in the French West Indies for decades, resulting in long term pollution, human health problems and social crisis. In addition to bacterial consortia and *Citrobacter* sp.86 previously described to transform chlordecone into three families of transformation products (A: hydrochlordecones, B: polychloroindenes and C: polychloroindenecarboxylic acids), another bacterium *Desulfovibrio* sp.86, showing the same abilities has been isolated and its genome was sequenced. Ring-opening dechlorination, leading to A, B and C families, was observed as previously described. Changing operating conditions in the presence of chlordecone gave rise to the formation of an unknown sulfur-containing transformation product instead of the aforementioned ones. Its structural elucidation enabled to conclude to a thiol derivative, which corresponds to an undocumented bacterial reductive sulfidation. Microbial experiments pointed out that the chlordecone thiol derivative was observed in anaerobiosis, and required the presence of an electron acceptor containing sulfur or hydrogen sulfide, in a confined atmosphere. It seems that this new reaction is also active on hydrochlordecones, as the 10-monohydrochlordecone A1 was transformed the same way. Moreover, the chlordecone thiol derivative called F1 was detected in several chlordecone contaminated mangrove bed sediments from Martinique Island, highlighting the environmental relevance of these results.

## Introduction

Chlordecone is a highly recalcitrant organochlorine pesticide that has been added to the Stockholm convention list of persistent organic pollutants in 2009. Several pest control products containing chlordecone (Kepone, Curlone) or a functionalized derivative (Kelevan) have been manufactured in the US, Brazil and France and applied in the Caribbean, Central America, West Africa, and Europe over an extended period of forty years^1^. To date, chlordecone has caused two major environmental disasters: (1) acute exposure of workers at the Hopewell (US) chlordecone production plant in 1975 and massive pollution of the James River, extending over 100 miles that lasted for decades^2^, (2) ongoing impregnation of the French West Indies (FWI) population due to extensive agricultural use of chlordecone from 1972 to 1993 that has resulted in long-term pollution of environmental compartments (soils, water resources, coastal areas) and subsequently of some local food production (vegetables, farmed animals, and seafood). Until now, no remediation strategy has been proven satisfactory in the FWI environment whereas the population has to deal with health troubles (increased risk of cancer, motor and cognitive development disorders in young children, premature births) and social difficulties (disappearance of local economy activities)^1^.

In this context, chlordecone microbial degradation has been the focus of several studies: some *Pseudomonas aeruginosa* sp. as well as mixed cultures isolated from the Hopewell area in the 1980s exhibited significant aerobic dechlorination ability leading to mono- and dihydrochlordecones transformation products (TPs)^3^. Later on, the conversion of chlordecone into unknown polar and nonpolar TPs in the presence of the anaerobic archaea *Methanosarcina thermophila* at 50 °C was reported^4^. More recently, two bacterial consortia (86 and 92), isolated from microbial enrichment cultures from chlordecone-contaminated soils and wastewater treatment plant sludge, were able to transform chlordecone, under anoxic conditions, at room temperature. Several TPs were identified and grouped into three families: hydrochlordecones (family A), polychloroindenes (family B) and polychloroindenecarboxylic acids (family C)^5,6^. TPs from families B and C that were predominant arose from a ring-opening dechlorination of the chlordecone bishomocubane cage. Metagenomic analysis of these consortia indicated the prevalence of *Citrobacter* and *Desulfovibrio* sp. genomes among the dozen of bacterial genomes sequenced. Two members of these consortia, *Citrobacter* sp.86 and *Citrobacter* sp.92 that were isolated, both degraded chlordecone resulting in the same TP profile. Reductive dehalogenases are key enzymes in halorespiration^7,8^ and could be indicative of a dehalogenation process. Here no gene sequences related to these enzymes were detected neither in the metagenomic data nor in the isolated *Citrobacter* genomes^5^.

The mechanistic degradation pathways and the exact role of each bacterium remains to be elucidated. In the past, several *Desulfovibrio* sp. or other sulfate-reducing bacteria have been reported in microbial cultures degrading chlorinated compounds^9–14^.

Herein, we report the isolation of *Desulfovibrio* sp.86 previously identified in chlordecone-degrading consortium 86^5^ and explore its capacity to transform chlordecone and other chlorinated derivatives. TPs from the B and C families as well TP A1 were detected in pure cultures of *Desulfovibrio* sp.86. Unexpectedly, other incubation conditions in presence of chlordecone gave rise to the formation an unknown sulfur-containing TP instead of the aforementioned TPs. The elucidation of its structure allowed us to conclude to a thiol derivative. To the best of our knowledge, it corresponds to an unprecedented bacterial reductive sulfidation. This thiol derivative was detected in several chlordecone-contaminated mangrove bed sediments from the Martinique Island.

## Results

### Isolation of *Desulfovibrio* sp.86

Isolation of *Desulfovibrio* sp.86 was achieved from the chlordecone-degrading consortium 86^5^ using sulfate-reducing conditions. As bacterial consortium 86, able to transform chlordecone, was obtained from an enriched mineral medium (MM) named MM + ^5^, supplemented with chlordecone, the main chemical composition was kept but electron donors and acceptors were modified. Several media formulations used to enrich sulfate-reducing bacteria utilize organic acids e.g. lactate, as carbon and energy sources (electron donor) and sulfate that is used as eletron-acceptor for growth^15–18^. In this context, pyruvate in the MM + liquid medium was replaced by lactate and sulfate was added (MMD liquid medium, see “Methods” section). The enrichment was spread on MMD agar and the brown vibrio-like bacterial colonies (observed under optical microscope) were purified further through two additional plate streakings (Fig. S1). An isolated bacterial strain was found to be identical to *Desulfovibrio* sp.86 from consortium 86 based on 100% identities of their 16S rRNA genes (1538 bp each).

### Genome analysis of *Desulfovibrio* sp.86

The whole genome consists of a single 3,464,070 bp circular chromosome. CheckM analysis^19^ performed with 61 genomes and 284 lineages indicates that the genome belongs to the Deltaproteobacteria and CheckM Completeness is 100% (zero essential marker missing). The average G + C content for the DNA is 58.06%. A total of 3,342 coding DNA sequences (CDSs) were predicted for the chromosome, 4 pseudogenes and 10 miscellaneous RNAs (misc-RNA), 3 rRNA operons, and 54 tRNA genes.

The three 16S rRNA genes of *Desulfovibrio* sp.86 are identical. Their best BLAST hits (NCBI) were from uncultured *Desulfovibrio* sp. clones from Microbial Fuel Cells such as MFC63A04 (Genbank accession number : FJ823865; coverage 98%; identity 99,87%)^20^. A phylogenetic tree using the available 16S rRNA of cultivable bacteria confirmed the similarities between *Desulfovibrio* sp.86 and *Desulfovibrio simplex* DSM4141 (Genbank 16S rRNA accession number: NR_117110; coverage 99%; identity 99, 22%; genomic sequence unavailable) (Fig. S2)^21^. At the genomic level, *Desulfovibrio* sp.86 ranks first with *Desulfovibrio desulfuricans* subsp. *desulfuricans* str. ATCC 27,774 genome (GenBank:NC_011883). Nevertheless, *Desulfovibrio* sp.86 shares only 1,408 genes (43%) with *D. desulfuricans* (over 80% amino acids identity and 80% alignment coverage). In addition, the average nucleotide identities (ANI) between *Desulfovibrio* sp.86 and sequenced *Desulfovibrio* genomes are lower than the 95% ANI cut-off value generally accepted for species delineation (Fig. S3)^22^. These results indicate that *Desulfovibrio* sp.86 is most probably a new species of the *Desulfovibrio* phylum. The presence of two superoxide dismutases and one catalase gene accounts for its relative oxygen tolerance. As expected, the *Desulfovibrio* sp.86 genome exhibits an extensive gene complement for sulfur metabolism, encompassing the sulfur respiration pathways with inorganic sources like sulfate, sulfite, bisulfite and tetrathionate as electron acceptors. Organic sulfur sources may be provided by fermentation products of sulfur biomass (sulfoquinovose of sulfoquinovosyl lipids) sulfoacetate, the sulfur non-proteogenic amino acid taurine via sulfoacetaldehyde, or alkanesulfonates^23^.

### *Desulfovibrio* sp.86 degrades chlordecone into known transformation products as well as an unexpected sulfur-containing compound

**Figure 1:**
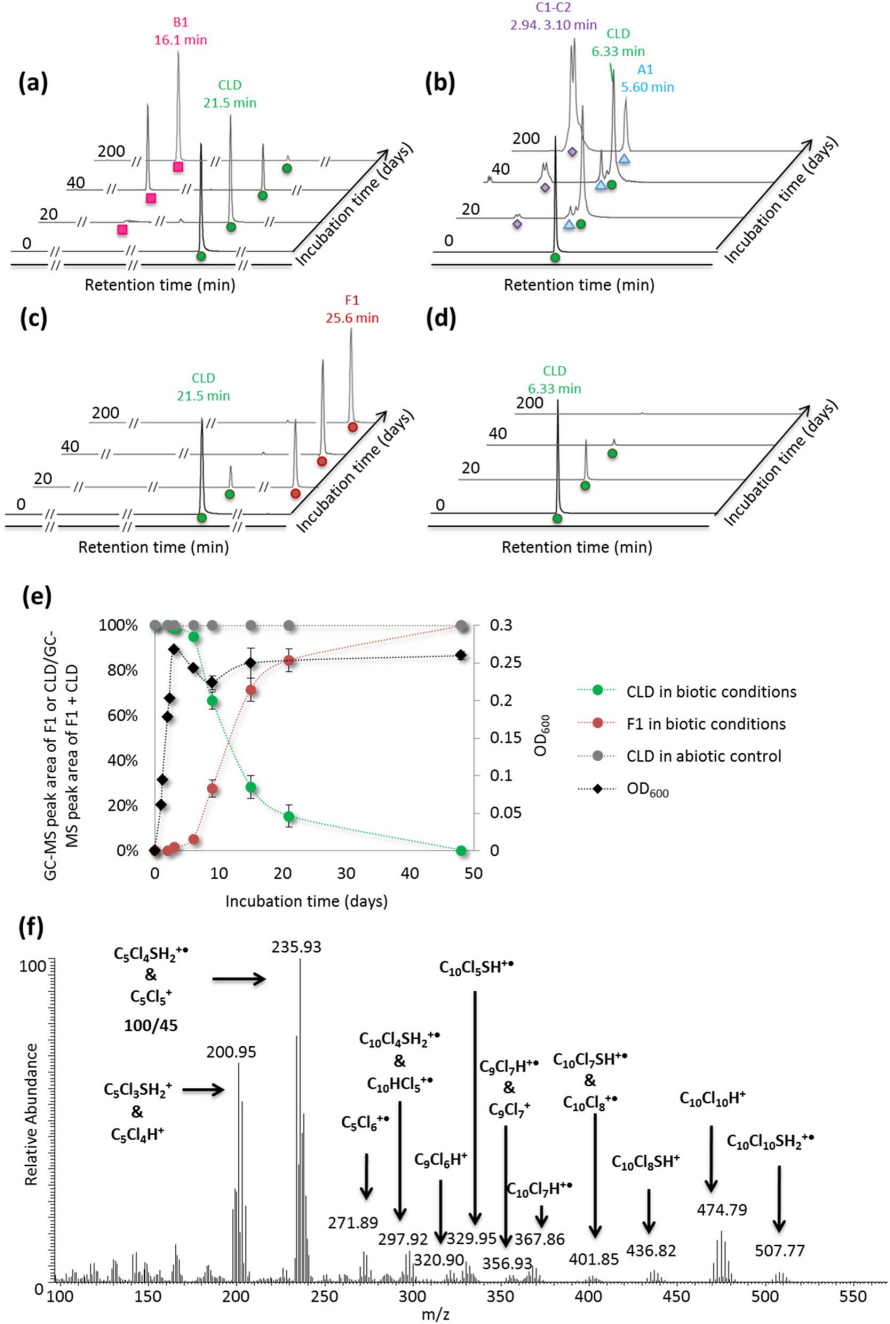
GC–MS and LC-HRMS monitoring, in full-scan mode (arbitrary unit), of chlordecone transformation by *Desulfovibrio* sp.86 in RA conditions (in glove box, anaerobiosis (N_2_/H_2_, 98/2, V/V), room temperature, open vials with a porous film, in MMD medium supplemented with 40 mg/L of chlordecone), and CA conditions (in oven, anaerobiosis (initially purged with N_2_/H_2_, 98/2, V/V), 30 °C, sealed vials, in MMD medium supplemented with 40 mg/L of chlordecone); and identification of TP F1. (**a**) GC–MS monitoring of chlordecone transformation by *Desulfovibrio* sp.86 in RA conditions. (**b**) LC-HRMS monitoring of chlordecone transformation by *Desulfovibrio* sp.86 in RA conditions. (**c**) GC–MS monitoring of chlordecone transformation by *Desulfovibrio* sp.86 in CA conditions. (**d**) LC-HRMS monitoring of chlordecone transformation by *Desulfovibrio* sp.86 in CA conditions. In each graph, green circles represent chlordecone, red circles F1, blue triangles 10-monohydrochlordecone A1, pink squares 2,4,5,6,7-pentachloroindene B1 and purple diamonds 2,5,6,7-tetrachloroindenecarboxylic acid and 2,4,5,6-tetrachloroindenecarboxylic acid C1–C2. In RA conditions traces of B3-B4 and C3–C4 TPs were also detected. (**e**) Chlordecone transformation over the time in CA conditions, OD_600_ corresponds to the optical density at 600 nm in biotic conditions. (**f**) GC mass spectrum of F1 TP and its interpretation.

The chlordecone-degrading ability of the isolated *Desulfovibrio* sp.86 strain was investigated using GC–MS (Gas Chromatography Mass Spectrometry) and LC-HRMS (Liquid Chromatography High Resolution Mass Spectrometry) techniques in growth conditions successfully applied for *Desulfovibrio* sp.86 isolation (MMD liquid medium). Na_2_S was used as reductant and an anaerobic N_2_/H_2_ (98/2; V/V) atmosphere was applied using a glove box system. These incubation conditions led to complete disappearance of the chlordecone signal in GC–MS and LC-HRMS, and resulted in similar GC–MS and LC-HRMS TP profiles as those obtained with *Citrobacter* sp.86^6^: monohydrochlordecone A1, pentachloroindene B1, tetrachloroindenes B3-B4 and polychloroindenecarboxylic acids C1-C2 and C3-C4 (Fig. 1a,b). In the glove box system, bacterial cultures were performed using 100 mL glass vials with a hydrophobic porous film to enable gas exchange and avoid contamination. This incubation condition was considered as “renewed atmosphere” condition (RA). In this system, the atmosphere was regularly renewed with N_2_/H_2_ (98/2; V/V) and the box was not thermostatically controlled so the temperature varied between 25 and 33 °C. To better control growth and degradation conditions, *Desulfovibrio* sp.86 cultures were placed in MMD medium using 100 mL glass vials, sealed with butyl rubber septa, in an oven at 30 °C. Sealed vials were initially purged with N_2_/H_2_ (98/2; V/V) gas to insure anaerobiosis. This incubation condition was considered as “confined atmosphere” conditions (CA).

**Figure 2:**
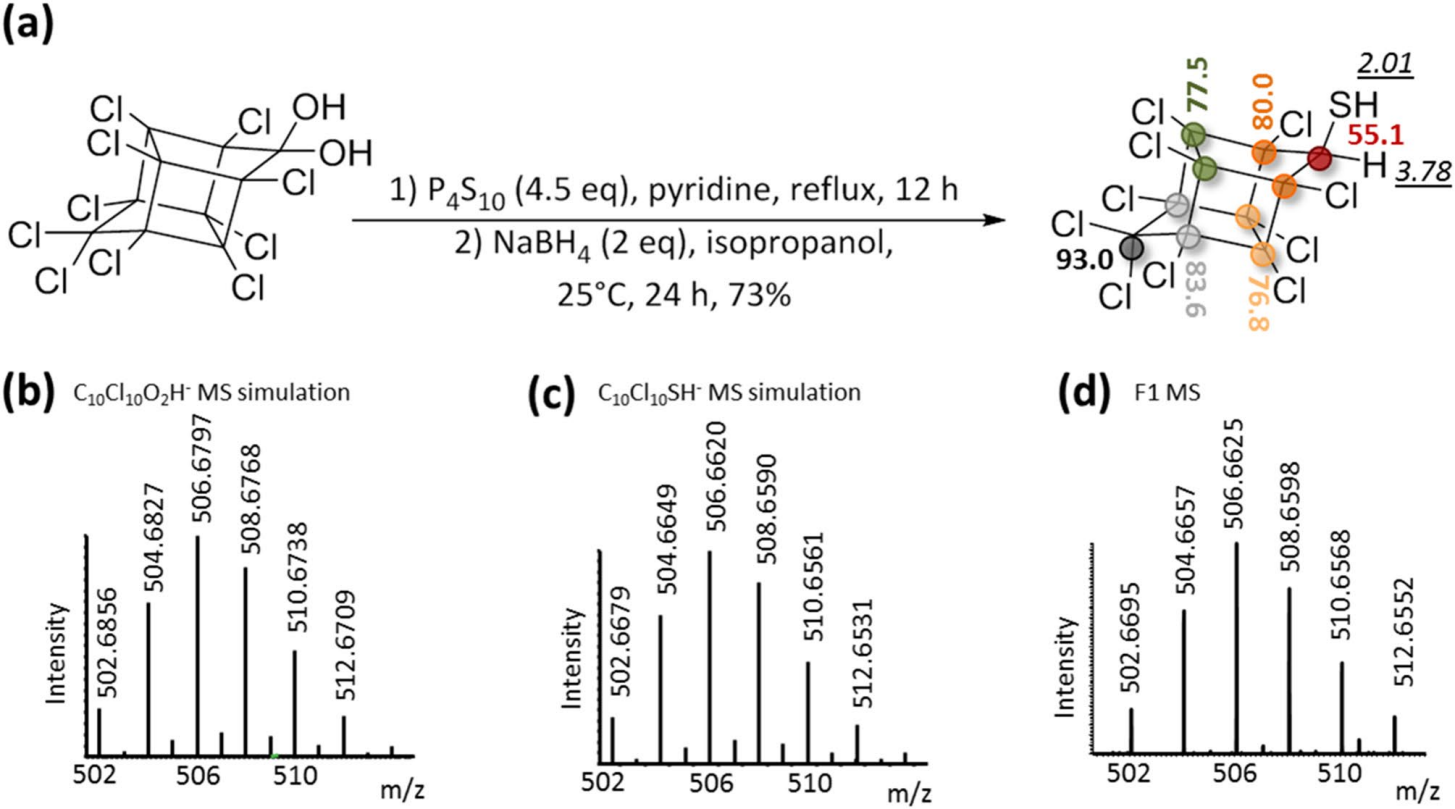
Synthesis of the chemical standard chlordecthiol and its complete characterization. (**a**) Chemical reaction scheme of chlordecthiol synthesis and NMR shifts in CD_2_Cl_2_: ^1^H NMR shift, in italic and underlined and ^13^C NMR shift, in bold; similarly colored circles indicate equivalent carbon atoms. (**b**) m/z simulation of C_10_Cl_10_O_2_H^-^, (**c**) m/z simulation of C_10_Cl_10_SH^-^, (**d**) extracted high resolution mass spectrum of synthetic chlordecthiol in negative mode (LC-HRMS).

After six-week incubation with chlordecone, the pesticide was no longer detectable by the same analytical techniques. Concomitantly, a single unknown chlorinated compound named F1 appeared. It was detectable only through GC–MS (25.6 min) (Fig. 1c). Like in the previously reported chlordecone degradation cultures^5,6^, the main chlordecone transformation appeared during the stationary phase (Fig. 1e). Since no visible chlorinated peak could be observed in LC-HRMS (Fig. 1d), we based the structural elucidation of F1 on the interpretation of GC–MS data (Fig. 1f). Assuming that the higher isotopic pattern centered at m/z 507.8 contained the molecular ion, we hypothesized two possible neutral formulae for F1, C_10_Cl_10_O_2_H_2_ and C_10_Cl_10_SH_2_. In-source fragments were highly similar to those observed in polychlorinated bishomocubane-based structures including chlordecone, hydrochlordecones, chlordecol and mirex^5,6,24^. Indeed, the isotopic patterns at m/z 201.0, 235.9 and 271.9 presumably arose from the known in-source bishomocubane retrocyclodimerization and corresponded to positively charged C_5_-fragments. Comparison with isotopic simulations limited the possibility to the following radical ions: [C_5_Cl_3+n_SH_2_]^+**·**^ [C_5_Cl_5+n_]^+**·**^ and [C_5_Cl_3+n_O_2_H_2_]^+**·**^, with n = 0,1. The latter bis-oxygenated generic formula turned to be highly unlikely since it required the presence of a gem-diol function or a gem-chlorohydrin moiety on the cyclopentenyl ring that had to resist the harsh GC chromatographic conditions (> 200 °C). Instead, we concluded that a sulfur moiety, i.e. a thiol function, was probably present on the bishomocubane polycycle. We proposed for F1 the structure depicted in Fig. 2a and suggested the name chlordecthiol, the sulfur analogue of chlordecol (chlordecone alcohol).

### Synthesis of the chlordecthiol standard and confirmation that transformation product F1 is identical to chlordecthiol

In order to confirm the structure of F1, the standard chlordecthiol was chemically synthesized and fully characterized. To achieve chemical reductive sulfidation of chlordecone, two steps were needed. The first one consisted in the conversion of the gem-diol function of chlordecone in equilibrium with the corresponding ketone form^20,25^ into the sulfur analog, i.e. gem-thiol/thiocarbonyl moiety. To perform this step, phosphorus-containing sulfur reagents are generally applied^26,27^. Here we used phosphorus decasulfide (P_4_S_10_) also called Berzelius reagent^27,28^ according to the protocol of Zaidi and coworkers who successfully synthesized camphorthiol^29^ (Fig. 2a). The second step consisted in the reduction of the gem-thiol intermediate using NaBH_4_. After purification, a white solid was obtained in an overall yield of 73% (Fig. 2a). 1D- and 2D-NMR analyses confirmed the chlordecthiol structure: (i) two ^1^H signals integrating each for one proton and coupled together (*δ* 3.78 ppm, d, *J* = 5.5 Hz and *δ* 2.01 ppm, d, *J* = 5.5 Hz), with the one at *δ* 3.78 ppm correlated to a ^13^C signal shifted downfield (*δ* 55.1 ppm) in HSQC experiment that perfectly account for the CH moiety next to the thiol function and (ii) a total of six visible ^13^C signals reflecting the plane of symmetry conserved in the present bishomocubane structure (Fig. 2a, Figs. S19–23). Although in microbial transformation, F1 could not be detected using LC-HRMS tool in the developed conditions, a concentrated sample of the synthetic standard chlordecthiol provided a significant signal. The presence of a sulfur atom and the expected neutral formula C_10_Cl_10_SH_2_ were confirmed (Fig. 2c,d), and the raw formulae C_10_Cl_10_O_2_H_2_ was ruled out (Fig. 2b). Lastly, GC–MS analysis demonstrated the perfect match (i.e., the same retention time of 25.6 min and same in-source mass spectra Fig. S6) between the synthetic standard and TP F1, thus definitely assigning it as chlordecthiol. Indeed, F1 represents the first member of a new family of chlordecone TPs possessing for the first time a sulfur atom.

### The ability of *Desulfovibrio* sp.86 to degrade chlordecone transformation products

Compounds A1, B1, C1-C2 and F1 representative of the four families of TPs possibly formed in presence of *Desulfovibrio* sp.86 were synthesized according to chemical protocols previously reported^6^ and herein developed for F1. Each of them was incubated in presence of *Desulfovibrio* sp.86 cultures in conditions that were shown to promote reductive sulfidation (sealed vial; CA conditions) or dechlorination with ring-opening (open vial; RA conditions). Dual GC–MS and LC-HRMS monitoring of all samples was performed.

**Figure 3:**
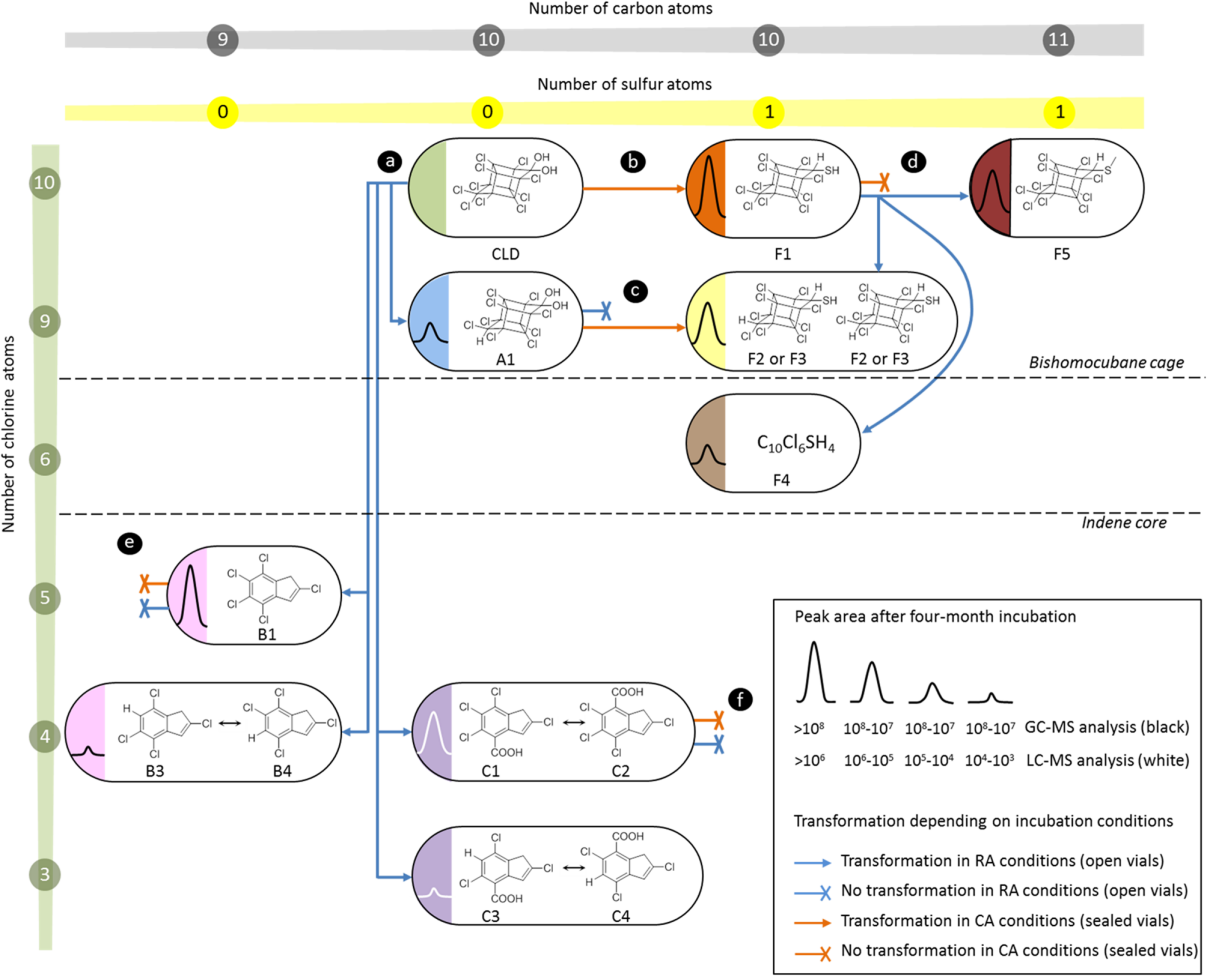
Fates of chlordecone transformation products with *Desulfovibrio* sp.86 depending on incubation conditions. (**a**) Transformation of chlordecone under RA conditions and (**b**) under CA conditions. Transformation of (**c**) A1, (**d**) F1, (**e**) B1 and (**f**) C1–C2.

After one-month incubation under CA conditions, 10-monohydrochlordecone A1 was completely transformed into two unknown chlorinated compounds barely separated using GC–MS (retention times: 24.3 min F2 and 24.5 min F3, Fig. 3, Figs. S7–S11). They showed an identical in-source mass spectrum that was highly similar to the F1 fragmentation pattern (Fig. S8). All detected in-source fragments turned to possess one chlorine atom less than their analogues in F1 mass spectrum. Compounds F2 and F3 were thus assumed to be diastereoisomers of 10-monohydrochlordecthiol (Fig. 3c). This was confirmed by the chemical synthesis of the two 10-monohydrochlordecthiol standards from 10-monohydrochlordecone A1 using the procedure previously applied for the synthesis of chlordecthiol F1 (Figs. S24–27). These chemical standards had the same retention times (24.3 and 24.5 min) and the same mass spectra compared to the biological F2 and F3 (Fig. S8). TPs B1, C1-C2 and F1 remained unchanged even after six-month incubation with *Desulfovibrio* sp.86 under CA conditions.

After six-month incubation under RA conditions, chlordecthiol F1 was partially converted into 10-monohydrochlordecthiols F2 and F3, and two other unknown chlorinated species detected using GC–MS, named F4 (retention time: 17.9 min) and F5 (retention time: 26.6 min) (Fig. 3d, Fig. S12). None of these two compounds gave any detectable signal in LC-HRMS. GC–MS analysis enabled to propose C_10_Cl_6_SH_4_ as raw formula for F4 (Fig. S14), and C_11_Cl_10_SH_4_ for F5 (Fig. S15). Methyl chlordecsulfide was chemically synthesized and fully characterized by NMR (Fig. S28–31). The perfect correspondence of GC retention times and GC mass spectra of methyl chlordecsulfide and biological F5 (Fig. S13) confirms its postulated identity. Noteworthy, the structure of F5 (Fig. 3d) represents a S-methylated form of F1. The exact nature of F4 remains to clear (see SI-Supplementary Text). All other TPs remained unchanged under RA conditions.

In summary, A1 (formed in RA conditions) underwent a reductive sulfidation just as chlordecone; under both incubation conditions polychloroindenes (B1 and C1-C2) did not seem to be degradable by *Desulfovibrio* sp.86, whereas F1 (produced under CA conditions) could be transformed under RA conditions (Fig. 3).

### Investigation of the incubation conditions leading to bacterial reductive sulfidation

To question the physico-chemical or physiological parameters influencing the transformations pathways of chlordecone in the cultures of *Desulfovibrio* sp.86, GC–MS monitoring (B1 and F1 presence as evidences of RA and CA conditions, respectively) was chosen.

Two sulfur-containing inorganic compounds, the reductant Na_2_S and the electron acceptor Na_2_SO_4,_ were present in the original MMD liquid medium. A first series of experiments in sealed vials with substitution of Na_2_S by other reducing agents, sulfured or not, i.e. cysteine and titanium(III) citrate (TiCi) led to a comparable level of chlordecthiol F1 (Table 1). Traces of TP B1 were also observed in all experiments, including Na_*2*_S, the positive control (Table 1).

**Table 1:** Influence of the reducing agent on chlordecone TP profile.

Culture parameters			*Desulfovibrio* sp.86	TPs (relative GC–MS peak area ratio TP/TPs + CLD)		
Atmosphere	Reducing agent	Electron acceptor		CLD	B1	F1
N_2_/H_2_ (98/2)	Na_2_S	Na_2_SO_4_	X	–	0.06	0.94
				1.00	–	–
	Cysteine		X	0.17	0.03	0.79
	TiCi (III)		X	0.17	0.03	0.79

GC–MS analysis of the two-month incubation extract: each TP peak area (full scan) was normalized to the sum of all peak areas (chlordecone and TPs).

A second set of experiments was designed using Na_2_S as reducing agent and alternative sulfur-based electron acceptors in sealed vials (Table 2). Use of 90% ^34^S-enriched inorganic sulfate resulted in a chlordecthiol product showing similar ^34^S-enrichment level (90% ^34^S-F1/10% F1, Fig. S16). GC–MS analysis of culture headspace also revealed the presence of H_2_^34^S and H_3_C^34^SH (Fig. S16), thus showing that *Desulfovbibrio* sp.86 produced H_2_S from sulfate. These gas were detected in the culture headspace in CA conditions, using MMD medium, whereas they were absent from the culture headspace in RA condition that corresponded to the glove box atmosphere (Fig. S17). Absence of sulfate did not inhibit *Desulfovibrio* sp.86 growth, however it resulted in formation of B1 (Table 2)^5^. The remplacement of sulfate by sulfite, bisulfite or thiosulfate led in all cases to the formation of chlordecthiol F1.

**Table 2:** Influence of the sulfur-based electron acceptor on chlordecone TP profile.

Culture parameters			*Desulfovibrio* sp.86	TPs (relative GC–MS peak area ratio TP/TPs + CLD)		
Atmosphere	Reducing agent	Electron acceptor		CLD	B1	F1
N_2_/H_2_ (98/2)	Na_2_S	Ø	X	0.51	0.49	–
		Na_2_SO_4_	X	0.05	–	0.95
		Na_2_SO_4_		1.00	–	–
		Na_2_^34^SO_4_	X	0.16	–	0.84
		Na_2_SO_3_	X	0.09	–	0.91
		NaHSO_3_	X	0.46	–	0.54
		Na_2_S_2_O_3_	X	0.10	–	0.90

GC–MS analysis of the two-month incubation extract: each TP peak area (full scan) was normalized to the sum of all peak areas (chlordecone and TPs).

A third series of experiments using jars investigated the effect of the nature of the gas phase in contact with the culture. Between the RA condition using open vials in glove box and CA condition using sealed vials in oven, several parameters differed (atmosphere nature and volume, temperature). Using a jar system including several vials, we selectively study the effect of atmosphere renewal on chlordecone transformation by *Desulfovibrio* sp.86 in MMD medium. In each case, open vials with hydrophobic porous films were placed in jars initially purged with a selected gas. All the jars were incubated at 30 °C. Some of them were flushed several times, to mimic the glove box system, whereas others were kept closed.

**Figure 4:**
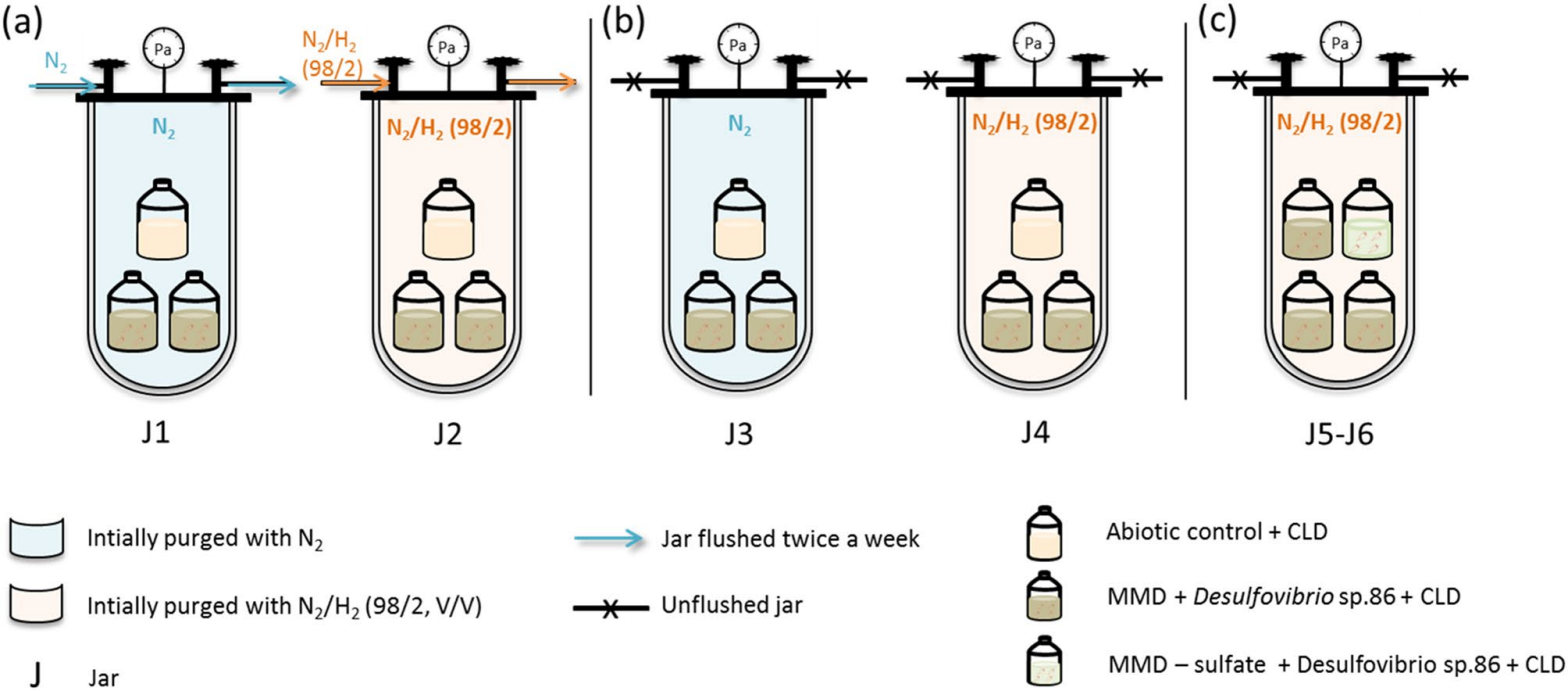
Schematic representation of gas-controlled experiments. (**a**) Flushed jars, (**b**) unflushed jars, (**c**) unflushed jars with sulfate-free *Desulfovibrio* sp.86 cultures.

Jars 1–4 contained two identical open vials filled with MMD, chlordecone and *Desulfovibrio* sp.86 inoculum, and one negative abiotic control. Among them, two jars were flushed twice a week, jar 1 with (N_2_/H_2_ (98/2; V/V)), jar 2 with N_2_ (Fig. 4a), whereas jars 3 and 4, initially purged with N_2_ and (N_2_/H_2_ (98/2; V/V)) respectively were left unflushed (Fig. 4b).

The last two jars (jar 5 and 6) contained three open vials filled with MMD, chlordecone and *Desulfovibrio* sp.86, plus one culture without sulfate. These jars were initially flushed with (N_2_/H_2_ (98/2; V/V)) and left unflushed for 2 months (Fig. 4c).

In parallel to the jars, two sealed vials filled with MMD without sulfate, chlordecone and *Desulfovibrio* sp.86 were flushed with extemporarily synthesized H_2_S (Fig. S4).

After two-month incubation at 30 °C, TP B1 was detected in vials placed in the flushed jars 1 and 2 (Table 3a), whereas TP F1 was only found in vials placed in unflushed jars 3 and 4 (Table 3b). No TP were present in the abiotic controls. In jars 5 and 6, F1 was present in both sulfate and sulfate-free *Desulfovibrio* sp.86 open cultures (Table 3c). In the same way, *Desulfovibrio* sp.86 sulfate-free cultures, flushed with H_2_S showed significant levels of F1 TP, whereas no transformation was observed in the negative control (Table 3d).

**Table 3:** Influence of gas atmosphere on chlordecone TP profile.

Experiment no.	Culture parameters			*Desulfovibrio* sp.86	TPs (relative GC–MS peak area ratio TP/TPs + CLD)		
	Atmosphere	Reducing agent	Electron acceptor		CLD	B1	F1
(a) J1	N_2_ flush	Na_2_S	Na_2_SO_4_	X	0.86	0.14	–
(a) J2	N_2_/H_2_ (98/2, V/V) flush			X	0.63	0.37	–
					1.00	–	–
(b) J3	N_2_			X	0.49	–	0.51
(b) J4	N_2_/H_2_ (98/2, V/V)			X	0.45	–	0.55
					1.00	–	–
(c) J5	N_2_/H_2_ (98/2, V/V)		Na_2_SO_4_	X	0.24	–	0.76
			Ø	X	0.55	–	0.45
(d)	H_2_S		Ø	X	0.36	–	0.64
					1.00	–	–

GC–MS analysis of the two-month incubation extract: each TP peak area (full scan) was normalized to the sum of all peak areas (chlordecone and TPs).

An additional chemical experiment was carried out to assess the influence of H_2_S on chlordecone transformation. Classic chemical protocol enabling B and C TPs formation, was applied (chlordecone, titanium citrate, vitamin B_12_, water). Under N_2_ atmosphere, B and C TPs were obtained, however under H_2_S atmosphere, only A1 was produced (Fig. S18).

These results clearly show that the formation of chlordecthiol F1 requires a closed incubation system with a reducing atmosphere. However H_2_ as the initial gas atmosphere is not mandatory whereas the presence of H_2_S is required. Chemically, the presence of H_2_S prevents from ring-opening dechlorination process.

### Environmental relevance of chlordecone reductive sulfidation

**Figure 5:**
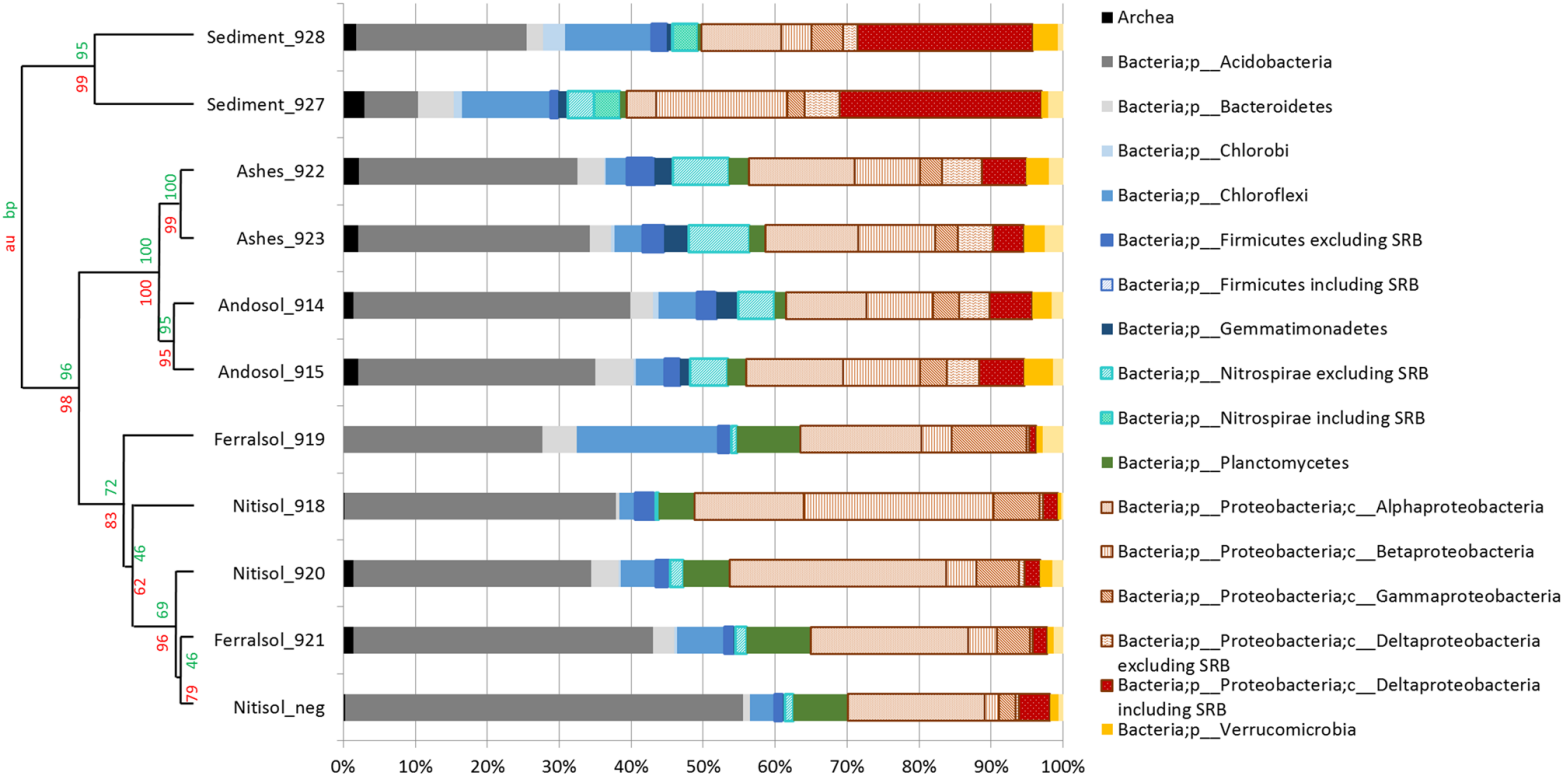
Dendrogram generated from hierarchical clustering of environmental samples according to the bacterial population diversity (from R package pvclust v. 1.3-2, https://doi.org/10.1093/bioinformatics/btl117). 200,000 sequences were taken for each environmental sample and normalized. The percentage of detected phyla was represented here with a focus on sulfate reducing bacteria from the *Deltaproteobacteria* class, Firmicutes and Nitrospirae phyla. In red is represented the unbiased (au) *p*-value and in green the bootstrap probability value (bp). SRB = Sulfate-reducing bacteria.

We reinvestigated our previous collection of the Martinique Island chlordecone-contaminated environmental samples (eight soils and two bed sediments) in which various levels of chlordecone TPs had been reported^6^. Among the novel sulfur-containing TPs herein reported, we found appreciable levels of F1 in the two bed sediments samples (927 and 928) at a concentration estimated around 50 μg/kg and 20 μg/kg of wet sediment, respectively (Table S1). In parallel, bacterial population diversity data issued from metabarcoding analysis of these samples (Fig. 5, Fig. S5) were processed to recover a hierarchical clustering of environmental samples according to their taxonomic composition^6^. It was noticed that sulfate-reducing bacteria were much more present in bed sediments than in the other compartments, as previously reported^30–32^.

## Discussion

To date, the mechanism of chlordecone microbial transformation by ring-opening dechlorination process has not been reported, nor has any associated enzyme activity been described. Bacteria from the genus *Desulfovibrio* are indeed known to be part of consortia able to dechlorinate molecules like aromatic hydrocarbons^32–35^. *Desulfovibrio* sp.86 is able to produce the same TPs of chlordecone as previously described^5,6^. However, the only other complete genome of a chlordecone degrading organism is from a *Citrobacter* species. The large divergence between these two bacterial species at the level of genus, lifestyle and metabolism precludes a straightforward differential genomic approach searching for common genes to understand ring-opening dechlorination process at the level of the pathway. Even if a parsimonious hypothesis could invoke the same mechanism for chlordecone ring-opening dechlorination process in both species, additional isolated species and genomes are welcomed to give clues to this question.

In this work, we also report on the identification, in the presence of *Desulfovibrio* sp.86, of another chlordecone transformation product issued from a reduction process, the sulfured molecule which we named chlordecthiol and which had not been described so far. The sulfur metabolism of bacteria from the genus *Desulfovibrio* is versatile, and encompasses the use of various inorganic sulfur sources that eventually can be reduced to H_2_S. The *Desulfovibrio* sp.86 gene content is consistent with the production of sulfide. In particular, under the experimental incubation conditions we used, reduction of sulfate from the mineral medium can be achieved by the sulfate and sulfite reductase genes.

The reductive sulfidation was also observed with the monohydrochlordecone A1 and then could be extrapolated to other hydrochlordecones. Remarkably, putting F1 in renewed atmosphere conditions essentially leads to sulfured analogs of F1 (F2 and F3), and the methylated derivative F5. This process generates a greater diversity of chlordecone TPs and extends the list of chlordecone derivatives that could be present in the environment. According to LC-HRMS analyses, F1 appeared less polar than chlordecone and chlordecol (Table S2). Indeed, its physico-chemical properties are likely to be different from those of chlordecone due to the replacement of the gem-diol fraction (chlordecone) by the thiol function (F1). The fate of F1 in the environment is therefore likely to be different in terms of biodegradability, stability, mobility to other environmental compartments and/or biomagnification along the food chain.

Sulfate reducing bacteria are frequently encountered in anaerobic environments like sediments where Fe(III) and sulfate dominate the pool of electron acceptor^34^. Thus, the environmental relevance of chlordecthiol detection that we report herein in chlordecone-contaminated sediments from the French West Indies may originate from microbial processes. Moreover, our work in laboratory condition indicates that at least some TPs of chlordecone are also prone to reductive sulfidation (hydrochlordecones). Even if at this stage a large set of questions related to their appearance, persistence, trophic food-chain transfer and toxicological profile can only be envisioned, our results open a new window on the secondary contamination by chlordecone TPs as well as possible clues to its natural elimination.

Additionally, the change in the TP profile observed in our experiments with *Desulfovibrio* sp.86 under various conditions of sulfur donor and atmosphere (confined or renewed in anaerobic conditions) could indicate biochemical pathways blockade and/or promotion triggered by H_2_S accumulation, leading to re-routing of chlordecone into this newly described reductive sulfidation instead of the previously reported ring-opening dechlorination TPs. At this stage, it is worth mentioning that all transformations of chlordecone with the consortium 86 had been conducted in open vials conditions, i.e. renewed atmosphere conditions. In a study related to dechlorination of trichlorofluoromethane by sulfate reducing bacteria, sulfide was proved to be inhibitory to the dehalogenation process^35^.

Some papers mentioned that H_2_S or HS^-^ can bind to metal centers and have an inhibitor effect on zinc and cobalt γ-class enzymes^36,37^. It was suggested that corrinoids could play a role in chlordecone ring-opening dechlorination conditions^5^ and broadly, vitamin B_12_, known to be a cofactor of reductive dehalogenases, can also intervene in the reductive dehalogenation processes as protein-free corrinoid^38–40^, in both biotic^41^ and abiotic settings^6,42^. In this work, we showed that vitamin B_12_ combined with titanium citrate and chlordecone under N_2_ atmosphere gave B and C families, whereas the same combination under H_2_S atmosphere only led to A1 formation (Fig. S18). The weakness of the bond strength of cobalt with H_2_O inducing a spontaneous reductive substitution of aquo − cobalamin(III) (Cbl(III) − H_2_O) by sulfide − cobalamin(III) (Cbl(III) − S^2−^) in the presence of S^2−^ was reported^43^. In the case of corrinoid implication in B and C families formation, it could be assessed that the presence of H_2_S could also be inhibitory and thus favored F1 by another mechanism.

Reductive sulfidation of ketones and aldehydes are possible using a large variety of chemical protocols, very distant from physiological conditions^27^. Several C-S bond formation are described, some mechanisms involve a glutathion-ester intermediate or a cysteine precursor to form thiol derivatives via nucleophilic reactions^44,45^, others use radical SAM enzymes^45^. Several enzymes known to catalyze sulfur addition reactions could take part in the reductive sulfidation of chlordecone. Among them thiolase, sulfurtransferase, sulfotransferase, sulfhydrylase and numerous Fe-S proteins are predicted in *Desulfovibrio* sp.86 genome. However, at this stage there is no clue enabling to suggest a mechanism and even to confirm an enzymatic activity toward chlordecone or TPs that were shown to undergo reductive sulfidation in *Desulfovibrio* sp.86 cultures. Further experiments will be necessary to consider if any of these processes are indeed operating with *Desulfovibrio* sp.86.

*Desulfovibrio* sp.86 is the first sulfate-reducing bacterium known to date that is able to transform chlordecone, and only the second isolated and sequenced bacterial species coping with this pesticide. In this work, we reported for the first time on microbial reductive sulfidation, which occured on substrates like chlordecone or hydrochlordecone. This raises the question of whether the microbial sulfidation process is restricted or not to these peculiar substrates, and whether it can be ascribed to the intrinsic sulfur-reducing ability of *Desulfovibrio* sp.86. In this respect, this new microbial strain, cultivated under our experimental conditions that promote reductive sulfidation, could provide a framework to investigate further this process with other natural or synthetic compounds. Finally, this study demonstrates that the way anaerobiosis is managed in the laboratory (e.g., renewed atmosphere in the glove box or atmosphere confined in sealed vials) is of critical importance for microbiological cultures as illustrated here by the change in the transformation pathways of chlordecone.

## Methods

### Chemicals and media

Chlordecone was obtained from Azur Isotopes (purity 98%). Na_2_S (≥ 98%), pyridine (99.8%), iodomethane (> 99%) and phosphorus pentasulfide (99%) were purchased from Sigma Aldrich. Titanium (III) citrate was prepared from titanium (III) chloride (> 12% in HCl) (Sigma Aldrich) and sodium citrate and neutralized with Na_2_CO_3_. Dichloromethane (DCM) and pentane (HPLC grade) were obtained from Fisher Chemical; cyclohexane (99.8%) and NMR solvents from Acros Organics; and acetonitrile (MeCN, LC-HRMS grade) and acetone (> 99.9%) from VWR Chemicals. Chemical products used for microbiological media were obtained from Sigma Aldrich. Chemicals and media used for chlordecone TPs are described elsewhere^6^.

### Analytics

GC–MS analyses were carried out using a Thermo Fisher Focus GC coupled to a single-quadrupole mass spectrometer (Thermo Fisher DSQ II). The instrument was equipped with a non-polar 30 m × 0.25 mm × 0.25 µm DB-5MS column (Agilent J&W) and a split/splitless injector. Ionization conditions and GC program were described elsewhere^5,6^.

HS-GC–MS (Head Space Gas Chromatography Mass Spectrometry) analyses were performed on a Thermo Fisher Trace 1300 coupled to ISQ 7000 VPI single quadrupole mass spectrometer. The instrument was equipped with a 30 m × 0.25 mm × 0.25 µm DB-624-UI column (Agilent J&W), a split/splitless injector and an automatic sampler TriPlus RSH coupled to a HeadSpace tool. For mass spectrometry (MS) analyses, the following standard working conditions were applied: electronic impact ionization, positive mode detection, ion source at 220 °C, detector voltage 70 eV, full scan mode m/z 33–300 (scan time 0.20 s). Injection and transfer line temperatures were set up at 200 °C, respectively 280 °C. 1 mL was injected each time with a filling speed of 10 min/mL, an injection speed of 10 mL/min and a penetration speed of 10 mL/s.

For H_2_S detection, vials were incubated for 1 min at 40 °C and sampled with a syringe at 40 °C. The split mode was applied (30 °C, flow rate at 16.7 mL/min, with a ratio of 33.4). The carrier gas was helium at 0.5 mL/min for 1 min followed by a gradient of 0.05 mL/min until reaching 1 mL/min (hold time 9 min). GC program was an isocratic at 24 °C for 20 min.

LC-HRMS analyses were carried out using a Dionex Ultimate 3000 LC system coupled to an LTQ-Orbitrap Elite mass spectrometer (Thermo Fisher Scientific) fitted with a heated electrospray ionization source (HESI) operating in negative ionization mode. Voltage optimization was described elsewhere^5^. Chromatographic separation was achieved using a Thermo Fisher Syncronis C18 column (50 mm length, 2.1 mm inner diameter, 1.7 µm particle size) and carried out at 30 °C with a flow rate of 0.5 mL/min using NH_4_OAc buffer (10 mM; pH 7 adjusted with NH_4_OH) as solvent A and MeCN as solvent B. The gradient started at 20% B for 1 min, followed by a linear gradient at 100% B for 7 min and remained 2 min at 100% B. The system returned to the initial solvent composition in 2 min and was re-equilibrated under these conditions for 2 min.

### Organochlorine extraction for microbiological culture monitoring

After homogenization of the liquid culture, 500 μL of the turbid solution were collected and extracted twice using 250 μL isooctane. The combined organic layers were then analysed through GC–MS analysis. When HS-GC–MS was required, 700 μL of cultures were sampled and put into a Chromacol 10-HSV vial of 10 mL (Agilent). For LC-HRMS analysis, 50 μL of the turbid solution were collected, mixed with 200 μL of acetonitrile/NH_4_OAc buffer (10 mM; pH 7 adjusted with NH_4_OH) (V/V, ¼) and filtered on 0.22 μm filters.

### Genomic DNA extraction, sequencing and analysis

DNA extraction was performed as described previously^5^.

Genome sequencing was performed mixing short and long reads (Illumina technology and Oxford Nanopore technology (ONT)). The Illumina and ONT data, around 280- and 20-fold coverage, respectively, were assembled using Unicycler (version: v0.4.6) (https://cab.spbu.ru/software/spades/) as described^46^. The annotation and research of putative orthologous relations between genomes defined as gene couples satisfying the bi-directional best hit (BBH) criterion or a blastP alignment threshold with a minimum of 35% sequence identity on 80% of the length of the smallest protein was performed using the Microscope platform^22^.

The final annotated genome of *Desulfovibrio* sp.86 is available in GenBank/ENA under the accession number LR738849 and project number PRJEB35237.

### Anoxic microbial incubations

In “renewed atmosphere” conditions, *Desulfovibrio* sp.86 cultures were incubated at 25–33 °C (room temperature) in a glove box (Unilab mBraun) under anaerobic conditions (N_2_/H_2_ (98/2; V/V)). They were carried out in MMD medium consisting of an enriched mineral medium MM previously described^5^ supplemented with lactate as carbon source (20 mM), yeast extract (1 g/L), Na_2_SO_4_ (7 mM) and Na_2_S as reducing agent (0.4 g/L). In the glove box, culture vials were open, equipped with a hydrophobic porous film (VWR, 114 μm thick, medical grade).

In “confined atmosphere” conditions, *Desulfovibrio* sp.86 cultures were incubated at 30 °C, in oven, under anaerobic conditions (N_2_/H_2_ (98/2; V/V)). They were carried out in MMD medium, in sealed culture vials, closed with butyl rubber septa.

*Desulfovibrio* sp.86 cultures were inoculated at the onset of an experiment with 0.5 mL pre-culture pre-grown in oven for 24 h.

During degradation reactions, the MMD medium was supplemented with 40 mg/L of chlordecone (from a solution of chlordecone at 200 mg/mL in dimethylformamide). Each experiment, tested condition or chlordecone degradation was done in duplicate, plus a negative control (without bacteria) in 100 mL glass serum vials containing 50 mL of mineral medium.

### Anoxic microbial degradation of chlordecone with different reducing agents

*Desulfovibrio* sp.86 cultures were incubated in CA conditions with chlordecone. They were carried out in MMD medium. And when indicated the reducing agent (Na_2_S, 0.4 g/L) was replaced by cysteine (1 g/L) or titanium citrate (0.38 mM).

### Anoxic microbial degradation of chlordecone with different electron acceptors

*Desulfovibrio* sp.86 cultures were incubated in CA conditions with chlordecone. They were carried out in MMD medium. And when indicated the electron acceptor (Na_2_SO_4_, 7 mM) was replaced by Na_2_^34^SO_4_, Na_2_SO_3_, NaHSO_3_ or Na_2_S_2_O_3_ (7 mM). Na_2_^34^SO_4_ was ^34^S enriched at 90%. For cultures containing Na_2_SO_3_ or Na_2_^34^SO_4_, the gas volume contained in the vial was analysed after two-month incubation by HS-GC–MS analysis.

### Gas-controlled chlordecone microbial degradation experiments

Six homemade anaerobic jars combined with a flushing system were used. Four jars contained two identical open 100 mL flasks filled with *Desulfovibrio* sp.86, chlordecone 40 mg/L, and MMD (50 mL), and one negative control flask with chlordecone (40 mg/L) and MMD (50 mL). Among these jars, two were flushed twice a week for 10 min, jar 1 with (N_2_/H_2_ (98/2; V/V)), jar 2 with N_2_, whereas jar 3 initially purged with N_2_ and 4, with (N_2_/H_2_ (98/2; V/V)) were left unflushed. Two extra jars (jars 5 and 6) contained three identical open 100 mL flasks filled with *Desulfovibrio* sp.86, chlordecone 40 mg/L, and MMD (50 mL), and one flask filled with *Desulfovibrio* sp.86, chlordecone (40 mg/L) and MMD pulling sulfate out (50 mL). These jars were initially purged with (N_2_/H_2_ (98/2; V/V)) and left unflushed. After two months incubation at 30 °C, all flasks were analyzed through GC–MS.

### Degradation of chlordecone TPs by *Desulfovibrio* sp.86

All degradation experiments were carried in duplicate 100 mL vials filled with 50 mL MMD inoculated with an actively growing *Desulfovibrio* sp.86 culture (1/100; V/V) in duplicate and an abiotic control was included. Chlordecone TPs were added to a final concentration of 80 μM in each vials from a stock solution. Cultures were incubated in a glove box or/and in the oven and monitored by GC–MS and/or LC-HRMS.

### Environmental samples extraction and analysis

Environmental samples origin and treatment were already described elsewhere^6^. They were collected on Martinique Island. Soil (2 andosol, 2 nitisol, 2 ferralsol soils and 2 ashes and 2 pumice stones) from the vicinity of the “Montagne Pelée” volcano and 2 bed sediment samples from Galion bay were taken from the 0–30 cm layer and conserved in glass boxes.

Dual chemical analysis of these samples (using GC–MS and LC–MS analysis) was already published elsewhere but without searching and taking into account the possible presence of sulfured TPs^6^. Samples were re-analysed and re-extracted according to the previously reported procedure^6^, to focus on sulfured chlordecone TPs. Each sample was processed in duplicate. For soils and sediments (10 g), 50 mL of milliQ water was added, followed by acidification to pH 1 with HCl (1 M) and vortexing. After decanting, the supernatant was extracted with DCM (10 × 50 mL) and the pellet washed twice with DCM (50 mL). Organic layers were pooled, concentrated *in vacuo*, and analyzed in duplicate injections by GC–MS (in dichloromethane). Soil sample from Martinique taken at a location known not to be contaminated with chlordecone (Nitisol_neg) was used as negative control, and was prepared and treated as mentioned above. GC–MS calibration curve was done using pure F1 compound in dichloromethane.

Soil biodiversity analysis was done on the previously described soil and bed sediment samples.The extraction protocol and the 16S rRNA gene pyrosequencing and analysis is described in Supplementary Information. Putative sulfate-reducing bacteria were assessed according to 16S rRNA sequences and according to the known sulfate-reducing bacteria already reported^17^. The list of families found in this analysis is given in Fig. S5. The dendrogram generated from hierarchical clustering of environmental samples was done using R package pvclust v. 1.3–2^47^.

### Chemical synthesis of F1 TP

Phosphorus pentasulfide (400 mg, 9.0 10^–4^ mol, 4.5 eq) was added to a solution of chlordecone (100 mg, 2.0 10^–4^, 1 eq) in pyridine (20 mL). The reaction mixture was stirred under N_2_, at reflux for 12 h. After cooling, 100 mL of pentane was added and the organic layer was successively washed with hydrochloric acid (2 M, 3 × 50 mL), distilled water and brine. Organic phase was dried over MgSO_4_, and concentrated under reduced pressure to give rise to an orange crude solid. To the crude solid, NaBH_4_ (22 mg, 6.0 10^–4^ mol, 3 eq) was added in 2-propanol (10 mL). The reaction mixture was stirred under N_2_, at room temperature, for 24 h. It was evaporated under reduced pressure and 10 mL of 5% H_2_SO_4_ was added. The acidic solution was extracted with pentane (3 × 50 mL). The organic layer was then evaporated under reduced pressure and F1 TP was purified by PLC (Preparative Layer Chromatography; Merck, PLC Silica gel, 2 mm, F_254_, 20 × 20) (cyclohexane/acetone 10:1; F1 retardation factor = 0.38). It was obtained as a white solid (73 mg; 1.4 10^–4^ mol; 73%).

### Chemical synthesis of F2–F3 TPs

Phosphorus pentasulfide (200 mg, 4.5 10^–4^ mol, 4.5 eq) was added to a solution of A1 (50 mg, 1.1 10^–5^, 1 eq) in pyridine (10 mL). The reaction mixture was stirred under N_2_, at reflux for 12 h. After cooling, 50 mL of pentane was added and the organic layer was successively washed with hydrochloric acid (2 M, 3 × 25 mL), distilled water and brine. Organic phase was dried over MgSO_4_, and concentrated under reduced pressure to give rise to an orange crude solid. F2-F3 TP were purified by PLC (Preparative Layer Chromatography; Merck, PLC Silica gel, 2 mm, F_254_, 20 × 20) (cyclohexane/acetone 10:1; F2-F3 retardation factors = 0.40); and obtained as white solids (13 mg; 2.8 10^–6^ mol; 26%).

### Chemical synthesis of F5 TP

To a solution of F1 (20 mg, 4.0 10^–5^ mol, 1 eq) and K_2_CO_3_ (52 mg, 4.0 10^–4^ mol, 10 eq) in acetone (50 mL), iodomethane (24 μL, 3.9 10^–4^ mol, 10 eq) was added. The reaction mixture was stirred under N_2_, in the dark, at room temperature, for 30 min. It was quenched with 50 mL of methanol and stirred for 15 min. After concentration under reduced pressure, the crude solid was purified by PLC (Preparative Layer Chromatography; Merck, PLC Silica gel, 2 mm, F_254_, 20 × 20) (cyclohexane/acetone 10:1; F5 retardation factor = 0.71); and F5 was obtained as a white solid (18 mg; 3.5 10^–5^ mol; 53%).

## Supplementary information

Supplementary information

## References

[1] Le Déaut, J. Y. & Procaccia, C. Les impacts de l'utilisation de la chlordécone et des pesticides aux Antilles: bilan et perspectives d'évolution. *OPECST* **487** (2009).

[2] Epstein SS (1978). Kepone-hazard evaluation. Sci. Total Environ..

[3] Orndorff SA, Colwell RR (1980). Microbial transformation of kepone. Appl. Environ. Microbiol..

[4] Jablonski PE, Pheasant DJ, Ferry JG (1996). Conversion of Kepone by Methanosarcina thermophila. FEMS Microbiol. Lett..

[5] Chaussonnerie S, Ugarte E, Saaidi P-L (2016). Microbial Degradation of a Recalcitrant Pesticide: Chlordecone. Front. Microbiol..

[6] Chevallier ML, Della-Negra O (2019). Natural chlordecone degradation revealed by numerous transformation products characterized in key French West Indies environmental compartments. Environ. Sci. Technol..

[7] Maymo-Gatell X, Chien Y, Gossett JM, Zinder SH (1997). Isolation of a bacterium that reductively dechlorinates tetrachloroethene to ethene. Science.

[8] Hug, L. A. *et al.* (2013) Overview of organohalide-respiring bacteria and a proposal for a classification system for reductive dehalogenases. *Philos. Trans. R. Soc. Lond. Ser. B Proc. Biol. Sci.* **368**, 20120322, 10.1098/rstb.2012.0322. 10.1098/rstb.2012.0322 PMC3638463 23479752

[9] Sun, B., Cole, J. R., Sanford, R. A. & Tiedje, J. M. Isolation and characterization of Desulfovibrio dechloracetivorans sp. nov., a marine dechlorinating bacterium growing by coupling the oxidation of acetate to the reductive dechlorination of 2-chlorophenol. *Appl. Environ. Microbiol.* **66**, 2408–2413. 10.1128/aem.66.6.2408-2413.2000 (2000). 10.1128/aem.66.6.2408-2413.2000 PMC110546 10831418

[10] Boyle AW, Phelps CD, Young LY (1999). Isolation from estuarine sediments of a Desulfovibrio strain which can grow on lactate coupled to the reductive dehalogenation of 2,4,6-tribromophenol. Appl. Environ. Microbiol..

[11] Lin XQ (2019). Accelerated microbial reductive dechlorination of 2,4,6-trichlorophenol by weak electrical stimulation. Water Res..

[12] Liu, J. & Haggblom, M. M. Genome-guided identification of organohalide-respiring deltaproteobacteria from the marine environment. *mBio*10.1128/mBio.02471-18 (2018). 10.1128/mBio.02471-18 PMC6299228 30563901

[13] Trueba-Santiso A (2020). Interspecies interaction and effect of co-contaminants in an anaerobic dichloromethane-degrading culture. Chemosphere.

[14] Wang, Q., Yang, M., Song, X., Tang, S. & Yu, L. Aerobic and Anaerobic Biodegradation of 1,2-Dibromoethane by a Microbial Consortium under Simulated Groundwater Conditions. *Int. Journal Environ. Rese. Public Health*10.3390/ijerph16193775 (2019). 10.3390/ijerph16193775 PMC6802363 31597267

[15] Postgate JR (1963). Versatile medium for the enumeration of sulfate-reducing bacteria. App. Microbiol..

[16] So CM, Young LY (1999). Isolation and characterization of a sulfate-reducing bacterium that anaerobically degrades alkanes. Appl. Environ. Microbiol..

[17] Muyzer G, Stams AJM (2008). The ecology and biotechnology of sulphate-reducing bacteria. Nature Rev. Microbiol..

[18] Ňancucheo, I. *et al.* Solid and liquid media for isolating and cultivating acidophilic and acid-tolerant sulfate-reducing bacteria. *FEMS Microbiol. Lett.*10.1093/femsle/fnw083 (2016). 10.1093/femsle/fnw083 27036143

[19] Parks DH (2015). CheckM: assessing the quality of microbial genomes recovered from isolates, single cells, and metagenomes. Genome Res..

[20] Borole AP, Mielenz JR, Vishnivetskaya TA, Hamilton CY (2009). Controlling accumulation of fermentation inhibitors in biorefinery recycle water using microbial fuel cells. Biotechnol. Biofuels.

[21] Yarza P (2013). Sequencing orphan species initiative (SOS): Filling the gaps in the 16S rRNA gene sequence database for all species with validly published names. Syst. Appl. Microbiol..

[22] Vallenet D (2020). MicroScope: an integrated platform for the annotation and exploration of microbial gene functions through genomic, pangenomic and metabolic comparative analysis. Nucl. Acids Res..

[23] Cook AM, Denger K (2002). Dissimilation of the C2 sulfonates. Arch. Microbiol..

[24] Alley EG, Layton BR, Minyard JP (1974). Identification of the photoproducts of the insecticides Mirex and Kepone. J. Agr. Food Chem..

[25] Wilson NK, Zehr RD (1979). Structures of some Kepone photoproducts and related chlorinated pentacyclodecanes by carbon-13 and proton nuclear magnetic resonance. The J. Org. Chem..

[26] Cortes-Santiago A, Navarrete-Lopez AM, Vargas R, Garza J (2017). Dissociation energy for the P2S2 ring in a family of thionation reagents and the corresponding chemical reactivity of separated species: a density functional theory analysis. J. Phys. Org..

[27] Murai, T. The Construction and Application of C=S Bonds. *Top. Curr. Chem. (Cham)* **376**, 31, 10.1007/s41061-018-0209-0 (2018). 10.1007/s41061-018-0209-0 29987439

[28] Ozturk T, Ertas E, Mert O (2010). A Berzelius reagent, phosphorus decasulfide (P4S10), in organic syntheses. Chem. Rev..

[29] Zaidi JH, Naeem F, Khan KM, Iqbal R, Ullah Z (2004). Synthesis of dithioacetals and oxathioacetals with chiral auxiliaries. Synth. Commun..

[30] Sanchez-Andrea, I., Stams, A. J., Hedrich, S., Nancucheo, I. & Johnson, D. B. Desulfosporosinus acididurans sp. nov.: an acidophilic sulfate-reducing bacterium isolated from acidic sediments. *Extremophiles* **19**, 39–47, 10.1007/s00792-014-0701-6 (2015) 10.1007/s00792-014-0701-6 25370366

[31] Luek JL, Thompson KE, Larsen RK, Heyes A, Gonsior M (2017). Sulfate reduction in sediments produces high levels of chromophoric dissolved organic matter. Sci. Rep..

[32] Jaekel U, Zedelius J, Wilkes H, Musat F (2015). Anaerobic degradation of cyclohexane by sulfate-reducing bacteria from hydrocarbon-contaminated marine sediments. Front. Microbiol..

[33] Song J (2019). The microbial community responsible for dechlorination and benzene ring opening during anaerobic degradation of 2,4,6-trichlorophenol. Sci. Total Environ..

[34] Kenneke, J. F. & Weber, E. I. Reductive dehalogenation of halomethanes in iron- and sulfate-reducing sediments. 1. Reactivity pattern analysis. *Environ. Sci. Technol.* **37**, 713–720, 10.1021/es0205941 (2003). 10.1021/es0205941 12636269

[35] Sonier DN, Duran NL, Smith GB (1994). Dechlorination of trichlorofluoromethane (CFC-11) by sulfate-reducing bacteria from an aquifer contaminated with halogenated aliphatic compounds. Appl. Environ. Microbiol..

[36] Innocenti, A., Zimmerman, S., Ferry, J. G., Scozzafava, A. & Supuran, C. T. Carbonic anhydrase inhibitors. Inhibition of the zinc and cobalt γ-class enzyme from the archaeon Methanosarcina thermophila with anions. *Bioorg. Med. Chem. Lett.* **14**, 3327–3331, 10.1016/j.bmcl.2004.03.101 (2004). 10.1016/j.bmcl.2004.03.101 15149700

[37] Cuevasanta E, Möller MN, Alvarez B (2017). Biological chemistry of hydrogen sulfide and persulfides. Arch. Biochem. Biophys..

[38] Bommer M (2014). Structural basis for organohalide respiration. Science.

[39] Renpenning J (2014). Combined C and Cl isotope effects indicate differences between corrinoids and enzyme (Sulfurospirillum multivorans PceA) in reductive dehalogenation of tetrachloroethene, but not trichloroethene. Environ. Sci. Technol..

[40] Payne KA (2015). Reductive dehalogenase structure suggests a mechanism for B12-dependent dehalogenation. Nature.

[41] Fetzner S, Lingens F (1994). Bacterial dehalogenases: biochemistry, genetics, and biotechnological applications. Microbiol. Rev..

[42] Schrauzer GN, Katz RN (1978). Reductive dechlorination and degradation of mirex and kepone with Vitamin B12. Bioinorg. Chem..

[43] Kyung D, Amir A, Choi K, Lee W (2015). Reductive Transformation of Tetrachloroethene Catalyzed by Sulfide-Cobalamin in Nano-Mackinawite Suspension. Ind. Eng. Chem. Res..

[44] Roland A, Schneider R, Razungles A, Cavelier F (2011). Varietal thiols in wine: discovery, analysis and applications. Chem. Rev..

[45] Naowarojna N (2018). Mini-review: ergothioneine and ovothiol biosyntheses, an unprecedented trans-sulfur strategy in natural product biosynthesis. Biochemistry.

[46] Fouteau, S. *et al.* Genome Sequence of Piezophilic Bacterium Desulfovibrio profundus Strain 500–1, Isolated from a Deep Sediment Layer in the Japan Sea. *Genome Announc.*10.1128/genomeA.01181-17 (2017). 10.1128/genomeA.01181-17 PMC5668535 29097459

[47] Suzuki R, Shimodaira H (2006). Pvclust: an R package for assessing the uncertainty in hierarchical clustering. Bioinformatics.

