# Supplementary material for: Transformation of the recalcitrant pesticide chlordecone by Desulfovibrio sp.86 with a switch from ring-opening dechlorination to reductive sulfidation activity
Source: Sci Rep. 2020 Aug 11;10:13545. doi: 10.1038/s41598-020-70124-9 (PMC7419502; doi:10.1038/s41598-020-70124-9)
Supplement: Supplementary file 1 — Supplementary information [file 41598_2020_70124_MOESM1_ESM.docx]

**Supplementary information for:**

Transformation of the recalcitrant pesticide chlordecone by Desulfovibrio sp.86 with a switch from ring-opening dechlorination to reductive sulfidation activity

*Oriane Della-Negra^a,1^, Sébastien Chaussonnerie^a,1^, Nuria Fonknechten^a^, Agnès Barbance^a^, Delphine Muselet^a^, Déborah E. Martin^a^, Stéphanie Fouteau^a^, Cécile Fischer^a^, Pierre-Loïc Saaidi^a,2,*^andDenis Le Paslier^a,2*^*

^a^Génomique Métabolique, Genoscope, Institut François Jacob, CEA, CNRS, Univ Evry, Université Paris-Saclay, 91057 Evry, France

^1^O. D. N. and S. C. contributed equally

^2^P-L. S. and D. L. P. contributed equally

*^*^To whom correspondence may be addressed: E-Mail: plsaaidi@genoscope.cns.fr or denis@genoscope.cns.fr.*

Summary of SI contents

**S1 Supplementary Methods 3**

**S2 Supplementary Text 5**

**S3 Supplementary Figures 8**

**S4 Supplementary Table 13**

**S5 Supplementary Chromatograms and Mass Spectra 14**

**S6 Supplementary NMR Spectra 26**

**S7 Supplementary References 39**

# S1 Supplementary Methods

**Chemical chlordecone degradation with vitamin B12, CiTi(III), under N_2_ or H_2_S atmosphere**

Titanium(III) citrate (2 mL, 1.25 × 10^−4^ mol, 32 equivalents) was added to chlordecone (2 mg, 3.9 × 10^−6^ mol, 1 equivalent) and vitamin B_12_ (2 mg, 1.3 × 10^−6^ mol, 0.3 equivalent). In the first case, H_2_O/ethanol 2/1 (6 mL) was degassed with N_2_, and the reaction mixture was stirred under N_2_ at room temperature for 80 min (Fig. S18a). In the second case H_2_O/ethanol 2/1 (6 mL) was degassed with H_2_S produced by P_4_S_10_ in contact with H_2_O, and the reaction mixture was stirred under H_2_S at room temperature for 80 min (Fig. S18b). A third reaction was done under N_2_ atmosphere, without CiTi(III) (Fig. S18c). And finally, a fourth reaction was done under N_2_ atmosphere, without vitamin B_12_ (Fig. S18d). All the three reactions were monitored by GC-MS analysis.

**Soil biodiversity analysis: 16S rRNA gene pyrosequencing and analysis**

DNA extraction of 9 soils was performed on 10 g of sample using the PowerMax Soil DNA Isolation Kit (MoBio) according to the manufacturer’s instructions. V4-V5 regions of the 16S rRNA gene were amplified with primers 515F (5’-GTGYCAGCMGCCGCGGTAA-3’) and 926R (5’-CCGYCAATTYMTTTRAGTTT-3’) then DNA libraries were sequenced using 250 bp paired end reads chemistry on a MiSeq Illumina sequencer. Illumina sequencing adapters and primer sequences were removed from the reads by an in-house designed software based on the FastX package. Reads were merged using the usearch v9.2.64 -fastq_mergepairs command^1^. The merged reads were cleaned, dereplicated and clusterized using the respective usearch v9.2.64 -fastq_filter, -derep_fulllength and -cluster_otus commands with default settings. The taxonomic assignation was performed on 200,000 sequences randomly selected with SortMeRNA v2.1^2^ using Greengenes database gg_13_5 (http://greengenes.secondgenome.com/downloads/database/13_5) with --id 0.97 and --coverage 0.97 parameters. 16S sequences from bacterial species present in the chlordecone-degrading consortia 86 and 92^3^ were added to the databases. The OTU table was generated using the usearch v9.2.64 -usearch_global command with -id 0.97.

# S2 Supplementary Text

**Structural indications of F4**

GC mass spectrum of compound F4 did not share any similarities with other known compounds. The molecular isotopic pattern led us to attribute C_10_Cl_6_SH_4_ as raw formulae and the observed in-source fragments were assigned as [C_10_Cl_5_SH_4_]^+^, [C_10_Cl_4_SH_4_]^+●^, [C_10_Cl_4_SH_3_]^+^, [C_10_Cl_3_SH_3_]^+●^, [C_10_Cl_3_SH_4_]^+^, arising from loss of HCl and/or Cl^●^. None C_5_ fragments, typical from chlordecone-like structures were observed, suggesting another structure for this compound. This compound could belong to another sulfured yet unknown TP family (Fig. S14).

**Structural elucidation of F5**

The GC-MS in-source fragments of compound F5 were more reminiscent of polychlorinated bishomocubane-based structures. The isotopic pattern centered at m/z 521.8 was assigned to C_11_Cl_10_SH_4_ as neutral formulae taking into account the presence of one sulfur atom in its precursor compound F1. It shared several positive ions with F1: [C_10_Cl_10_H]^+^, [C_10_Cl_8_]^+●^, [C_10_Cl_7_H]^+●^, [C_10_Cl_5_SH]^+●^, [C_10_HCl_5_]^+●^, [C_5_Cl_4_H]^+^, [C_5_Cl_5_]^+^ implying that these two compounds might exhibit a common core structure. Additional isotopic patterns attributed to [C_6_Cl_4_SH_4_]^+●^ (m/z = 247.9) and [C_6_Cl_3_SH_4_]^+^ (m/z = 212.9) were indicative of a methylated thiol moiety. All these observations are consistent with a possible methyl chlordecsulfide structure for compound F5 (Fig. S15). To confirm this hypothesis, the chemical synthesis of methyl chlordecsulfide standard was performed. Methylation of chlordecthiol F1 was carried out using classical procedure using iodomethane as methyl donor. After 15 min reaction time, GC-MS analysis showed the concomitant disappearance of F1 and the formation of a single product that perfectly matches the retention time and the in-source mass spectrum of compound F5 (Fig. S13). After purification, complete structure elucidation was achieved using several NMR experiments. ^1^H NMR spectrum showed four singlets, two corresponding to F5 and two others that could be attributed to the oxidized form of F5 (-S-S- or O=S=O or S=O). Among the singlets of interest, one was at *δ* 3.94 ppm (s, 1H) and another one at *δ* 2.33 ppm in the alkyl domain, integrating for 3H. In ^13^C NMR, six signals were highly similar to those observed in chlordecthiol ^13^C spectrum and accounted for the sulfidated bishomocubane structure. The last visible ^13^C signal at *δ* 17.0 ppm referred to the methyl group branched to the sulfur atom. Bi-dimensional heteronuclear NMR experiments (HSQC and HMBC) showed the expected ^1^J(C-H) and ^3^J(C-H) correlations between ^13^C and ^1^H atoms surrounding the sulfur atom (Fig. S28-31). We thus assigned TP F5 to the methyl chlordecsulfide structure. The impurity invisible in GC-MS could be assigned to the oxidized form, most probably the 0=S derivative, as similar singlets were observed in ^1^H NMR experiment (* *4.01 ppm, s 1H and * *2.91 ppm, s, 3H) and in ^13^C NMR experiment with the methyl group associated to a carbon shift of 38.8 ppm involving a deshielding effect.

# S3 Supplementary Figures


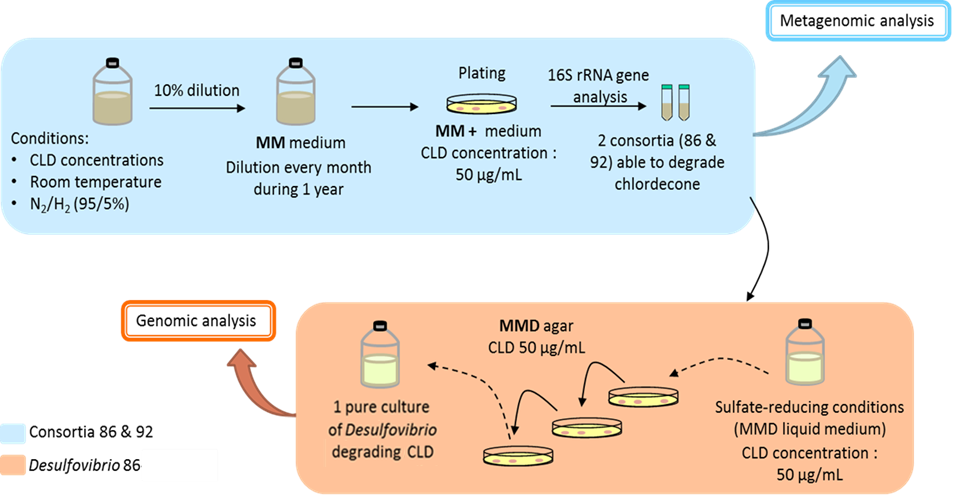


Figure S1: Isolation of *Desulfovibrio* sp.86 pure culture. MM (mineral medium) referred to a medium used in Chaussonnerie et al., 2016. MMD was defined in “Methods” section.


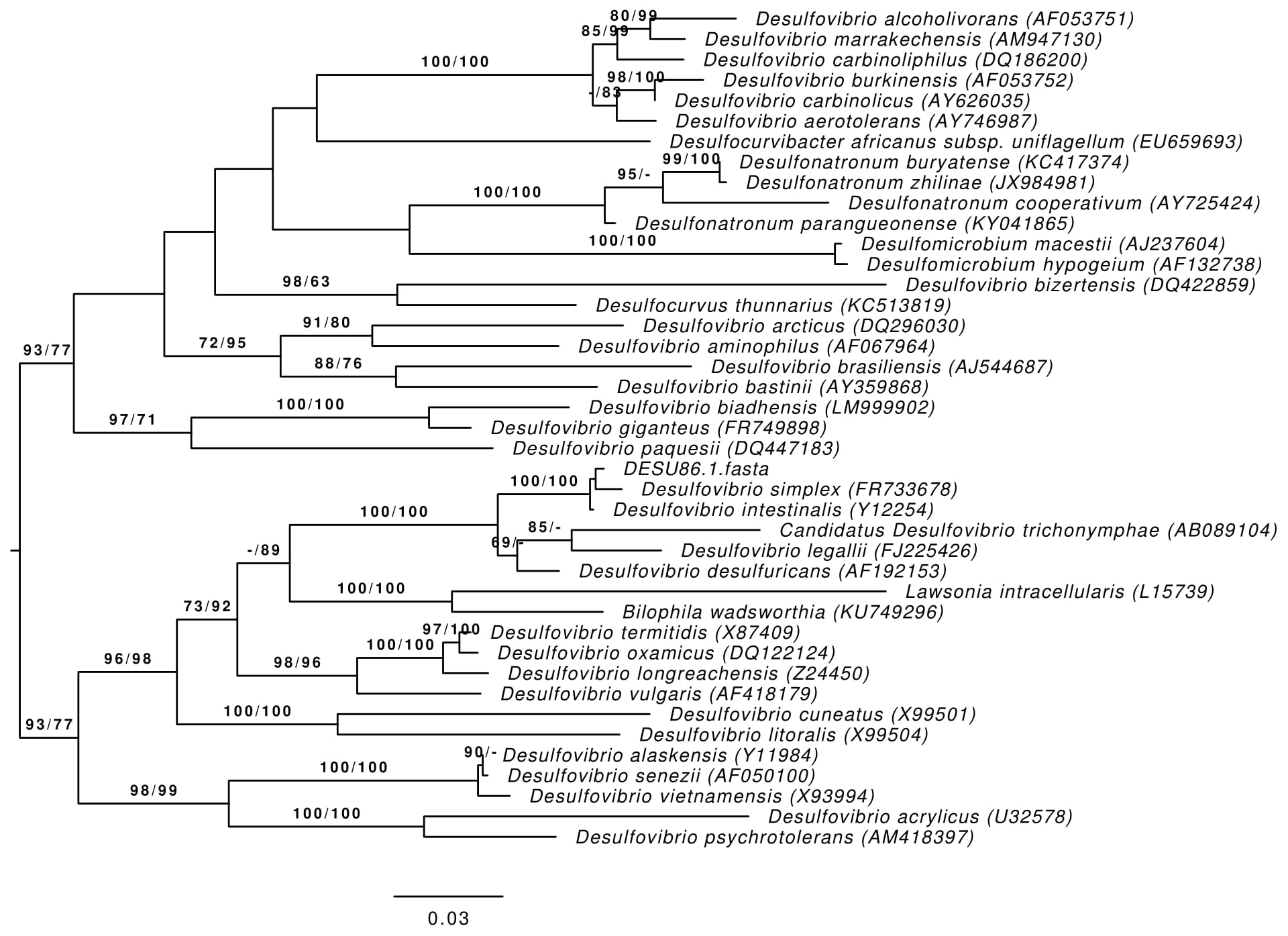


Figure S2: Maximum likelihood (ML) phylogenetic tree of 16S rRNA gene sequences showing the position of strain *Desulfovibrio* sp.86 relative to other species of the genus *Desulfovibrionaceae*. The branches are scaled in terms of the expected number of substitutions per site. The numbers above the branches are support values from ML (left) and maximum parsimony (right) bootstrapping, based on 1000 replications. Bar indicates 3.0 % sequence divergence.


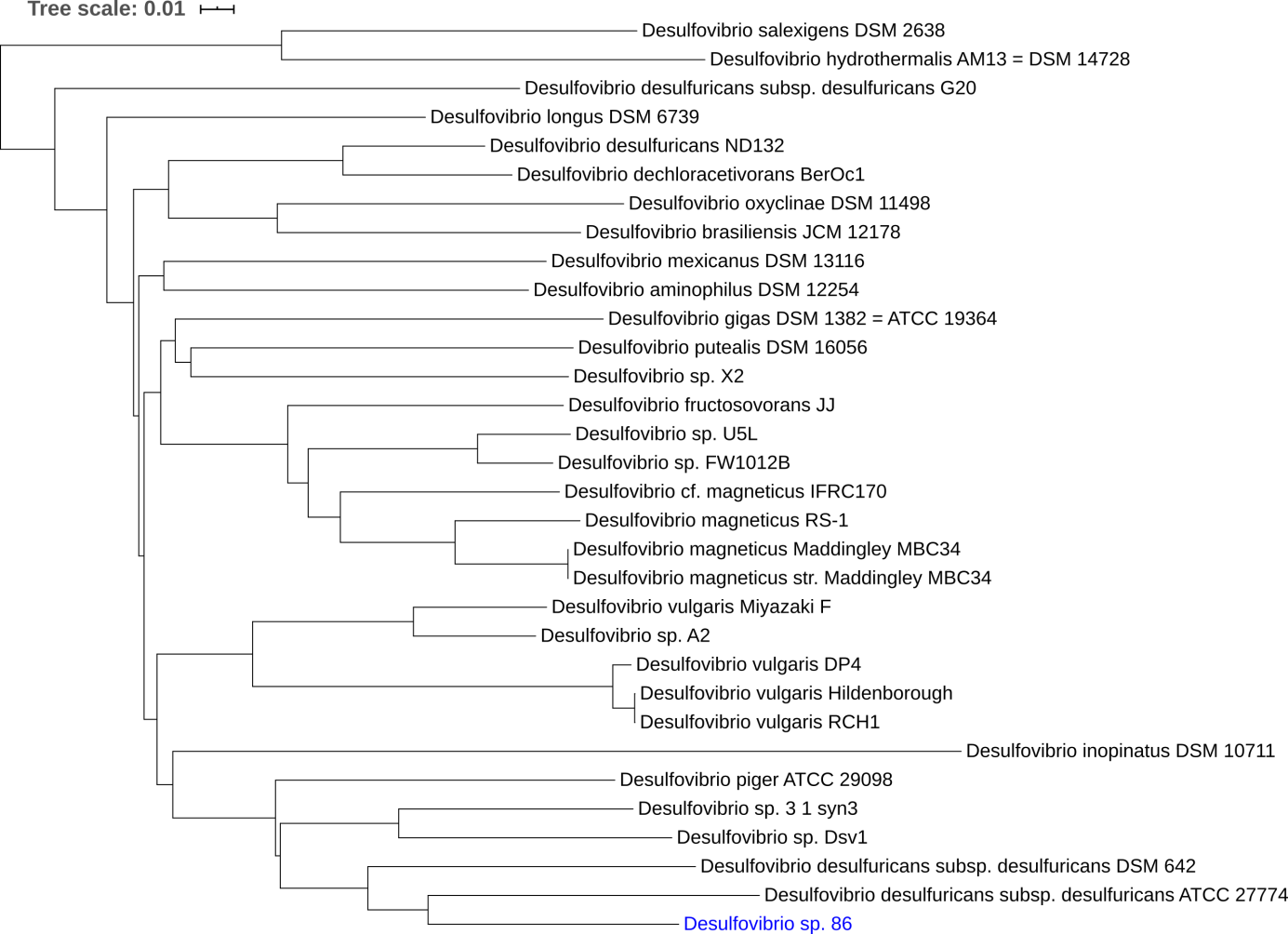


Figure S3: Clustering tree based on genomic similarity computed with MASH on MicroScope platform^4, 5^, displayed with iTOL^6^.


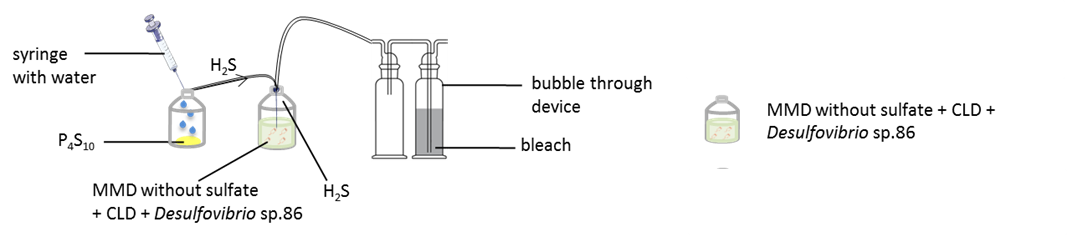


Figure S4: H_2_S is extemporaneously produced by the hydrolysis of P_4_S_10_ and directly flushed into the *Desulfovibrio* sp.86 cultures including sulfate-free MMD medium and chlordecone.


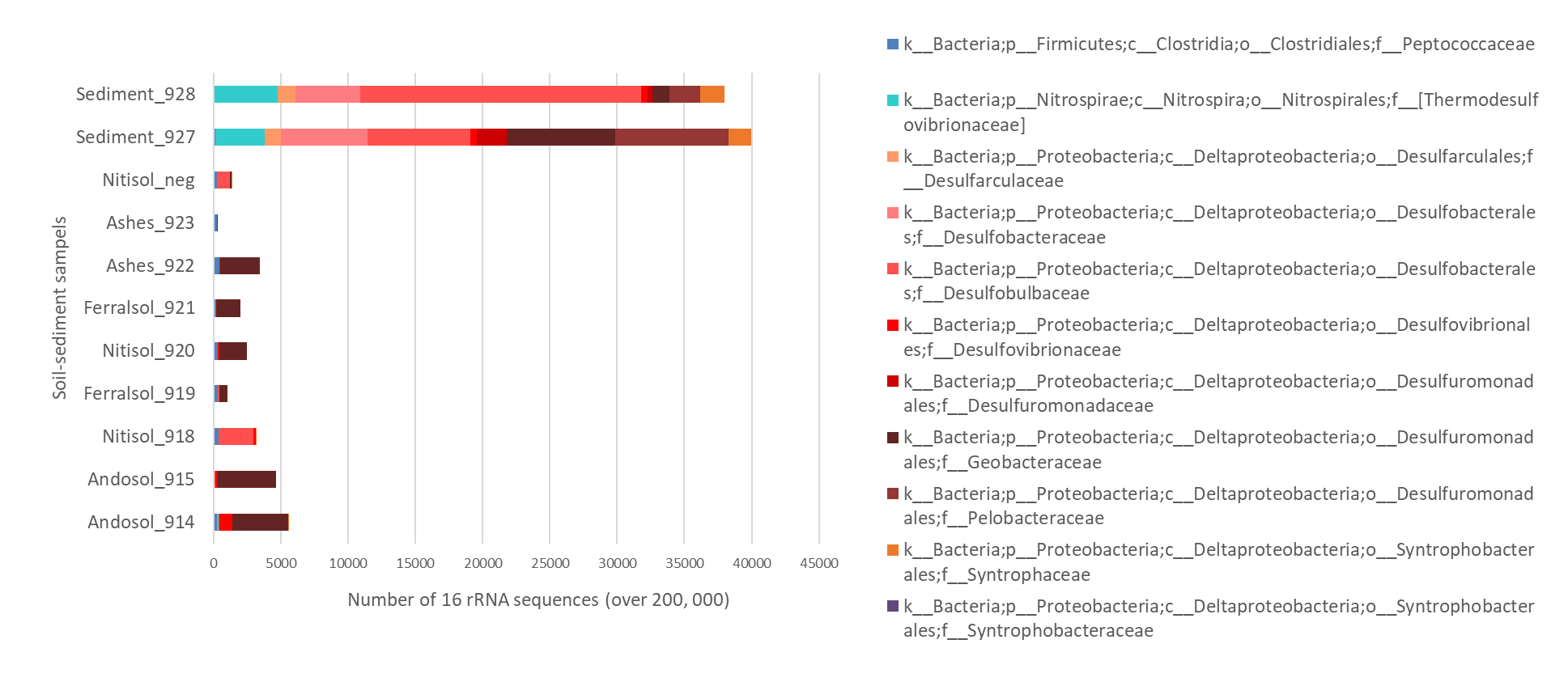


Figure S5: Abundance of putative sulfate-reducing bacteria in Martinique Island environmental samples based on the analysis of 16S rRNA partial sequences. The histogram represents the number of sequences assigned to sulfate-reducing bacteria (SRB), (with differential coloring for the various families) over 200,000 randomly-sorted sequences. All sampling locations, with the exception of Nitisol_neg, have been contaminated with chlordecone.

# S4 Supplementary Table

| Sample ID | F1 peak area | Concentration (mg/kg) | | Mean concentration (mg/kg) | Standard deviation |  |
| --- | --- | --- | --- | --- | --- | --- |
| 927-1 | 2.21E+06 | 0.036 | 0.047 | | 0.006 |  |
| 927-2 | 3.12E+06 | 0.048 |  |  |  |  |
| 927-3 | 3.53E+06 | 0.054 |  |  |  |  |
| 927-4 | 3.24E+06 | 0.050 |  |  |  |  |
| 928-1 | 1.47E+06 | 0.025 | 0.021 | | 0.005 |  |
| 928-2 | 1.62E+06 | 0.027 |  |  |  |  |
| 928-3 | 7.79E+05 | 0.016 |  |  |  |  |
| 928-4 | 8.67E+05 | 0.017 |  |  |  |  |

Table S1 : F1 calibration curve and F1 concentration in two sediment samples (927 and 928) from Martinique Island.

| Compounds | LC-HRMS retention time (min) | TLC retention factor in hexane/acetone (4/1, V/V) |
| --- | --- | --- |
| Chlordecone | 6.33 | 0.28 |
| Chlordecol | 7.10 | 0.54 |
| Chlordecthiol | 7.21 | 0.38 |

Table S2: Retention times of selected compounds using LC-HRMS method described in Methods section, obtained on a Thermo Fisher Syncronis C18 column (50 mm length, 2.1 mm inner diameter, 1.7 µm particle size) and TLC (Thin Layer Chromatography) retention factors obtained in hexane/acetone (4/1, V/V).

# S5 Supplementary Chromatograms and Mass Spectra


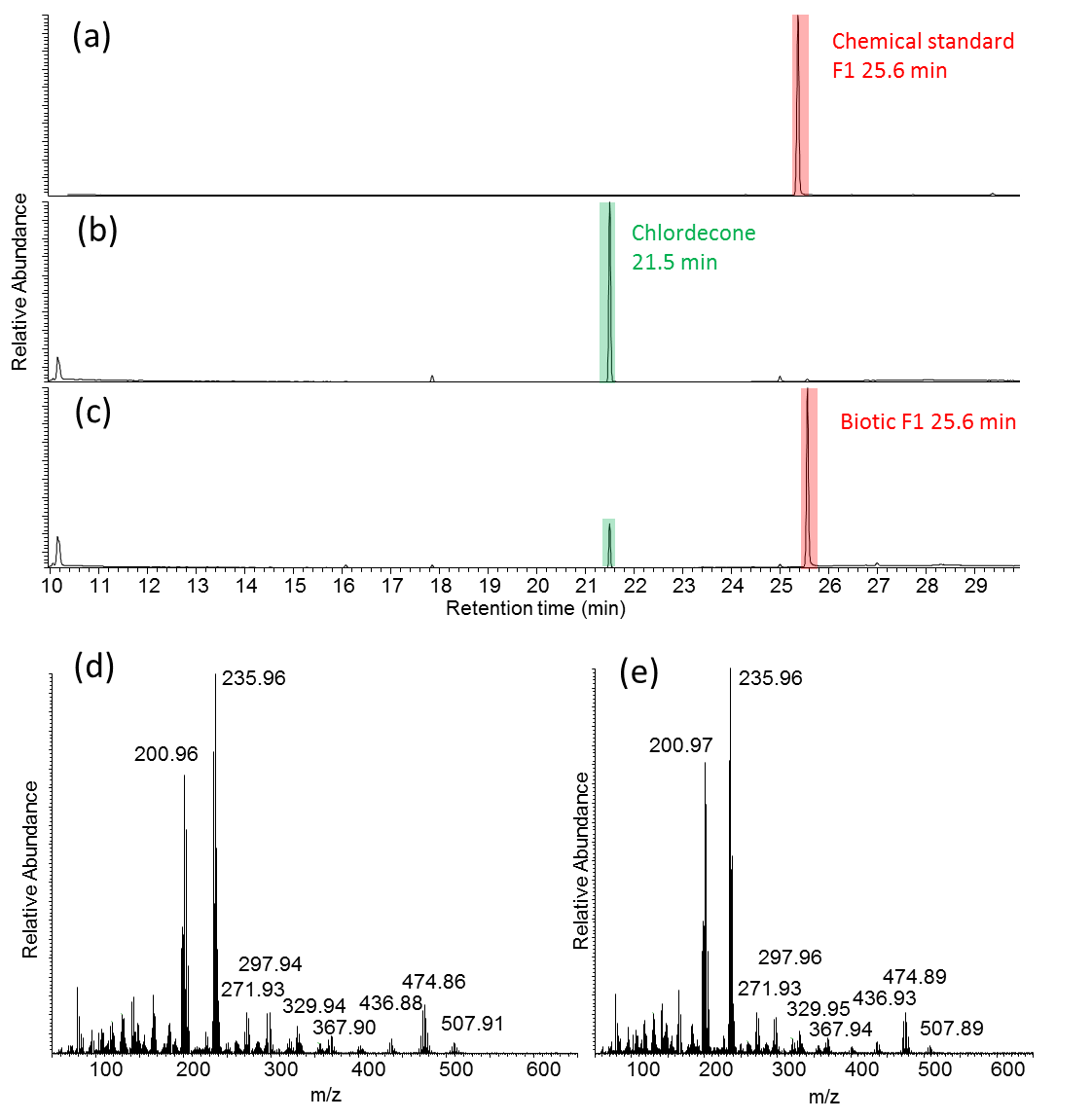


Figure S6: GC-MS identification of biological F1 with the chemical standard chlordecthiol. (a) GC-MS chromatogram (full scan acquisition) of the chemical standard chlordecthiol. (b) GC-MS chromatogram (full scan acquisition) of chlordecone. (c) GC-MS chromatogram (full scan acquisition) of a *Desulfovibrio* sp.86 culture incubated in CA condition, using MMD medium supplemented with chlordecone, after an incubation period of 20 days. (d) Mass spectrum of the chemical standard F1 from GC-EI-MS (positive mode) analysis. E-Mass spectrum of the microbial F1 from GC-EI-MS (positive mode) analysis.


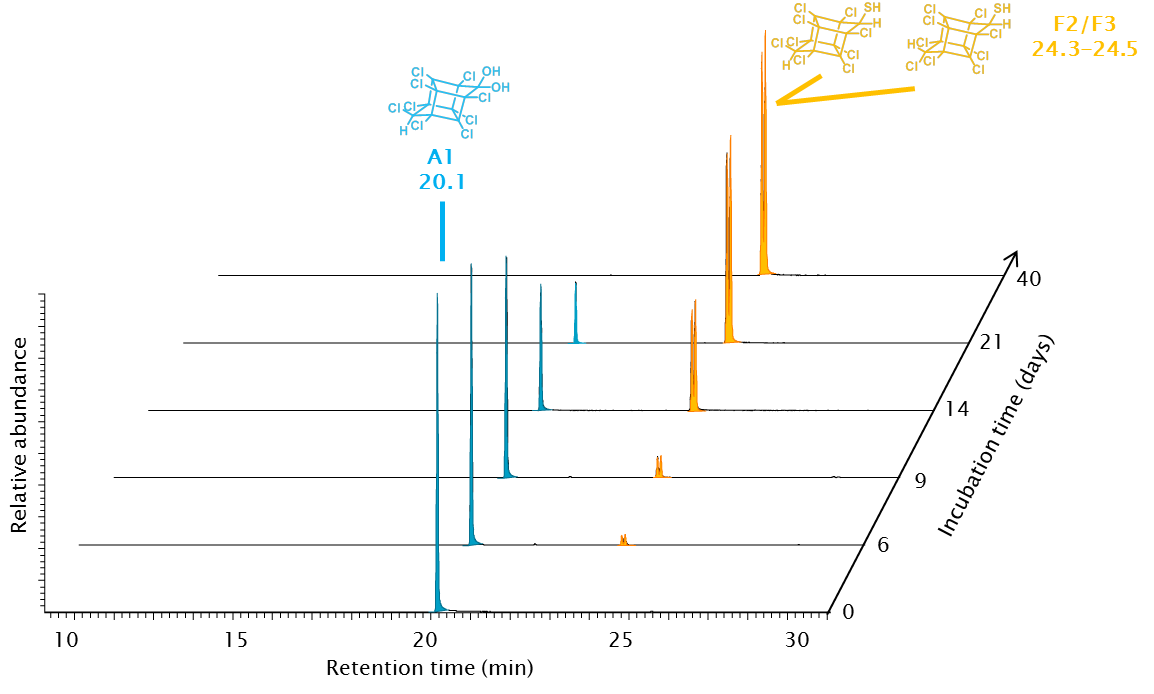


Figure S7: GC-MS monitoring of A1 transformation by *Desulfovibrio* sp.86 in sealed vials, MMD medium and N_2_/H_2_ (98/2, V/V) atmosphere (full scan acquisition).


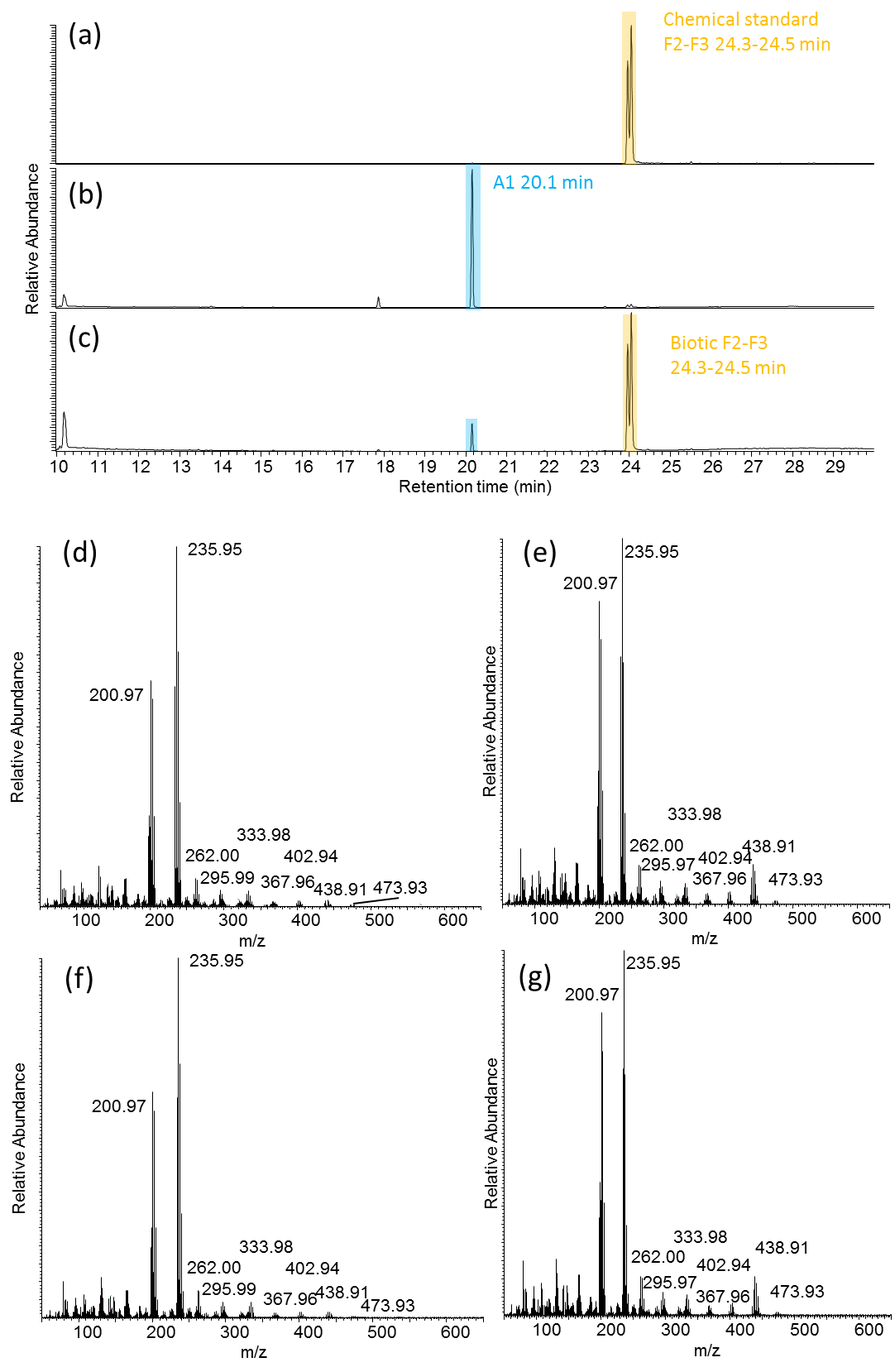


Figure S8: GC-MS analytical validation of F2/F3 standards. (a) GC-MS chromatogram (full scan acquisition) of chemical standards F2/F3. (b) GC-MS chromatogram (full scan acquisition) of A1. C-GC-MS chromatogram (full scan acquisition) of a *Desulfovibrio* sp.86 culture incubated in CA condition, using MMD medium supplemented with A1, after an incubation period of 20 days. (d, e) Mass spectra of chemical standards F2/F3 respectively from GC-EI-MS (positive mode) analysis. (f, g) Mass spectra of microbial F2/F3 respectively from GC-EI-MS (positive mode) analysis.


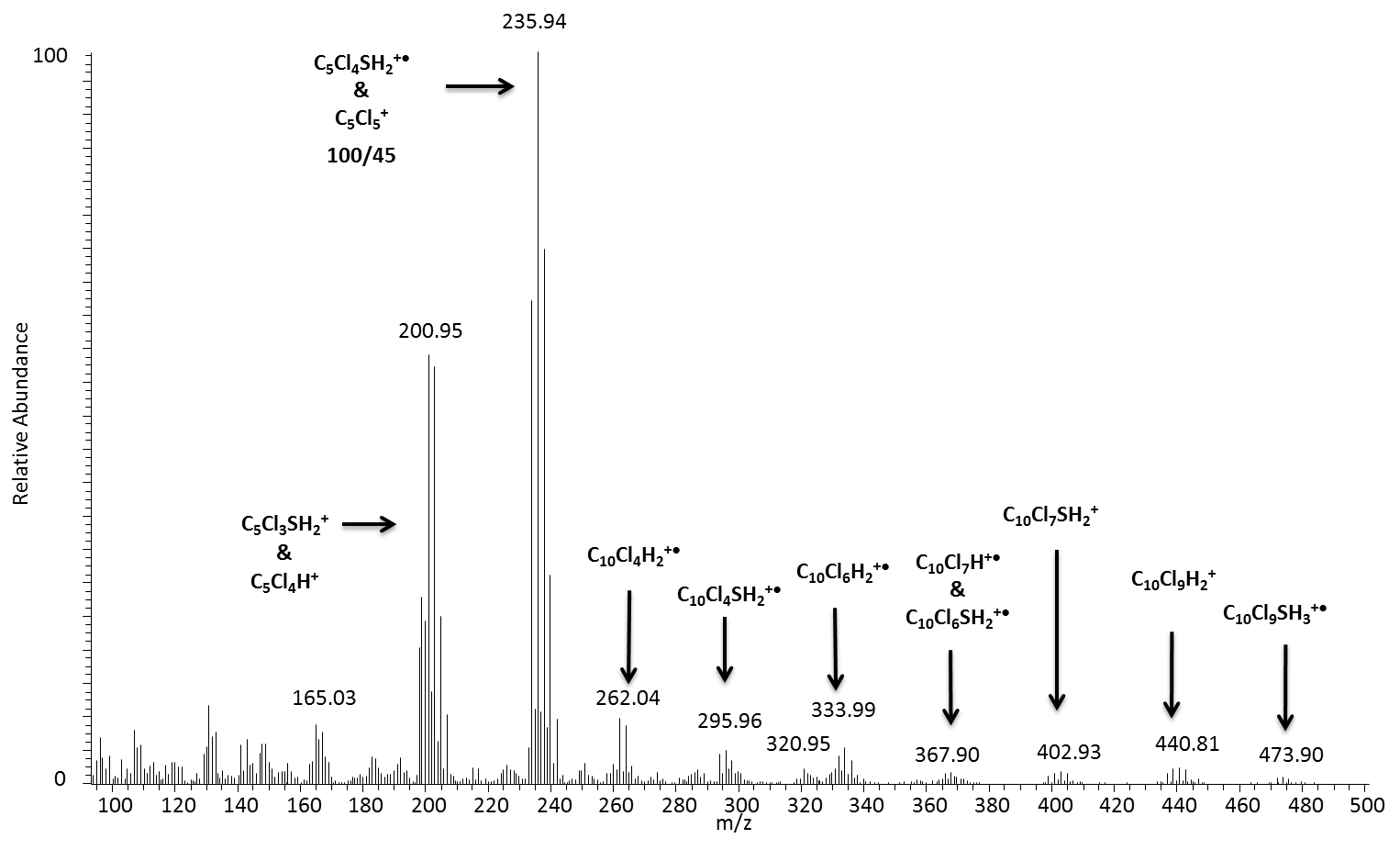


Figure S9: Mass spectrum of F2 from GC-EI-MS (positive mode) analysis and proposed in-source fragment ions assignment.


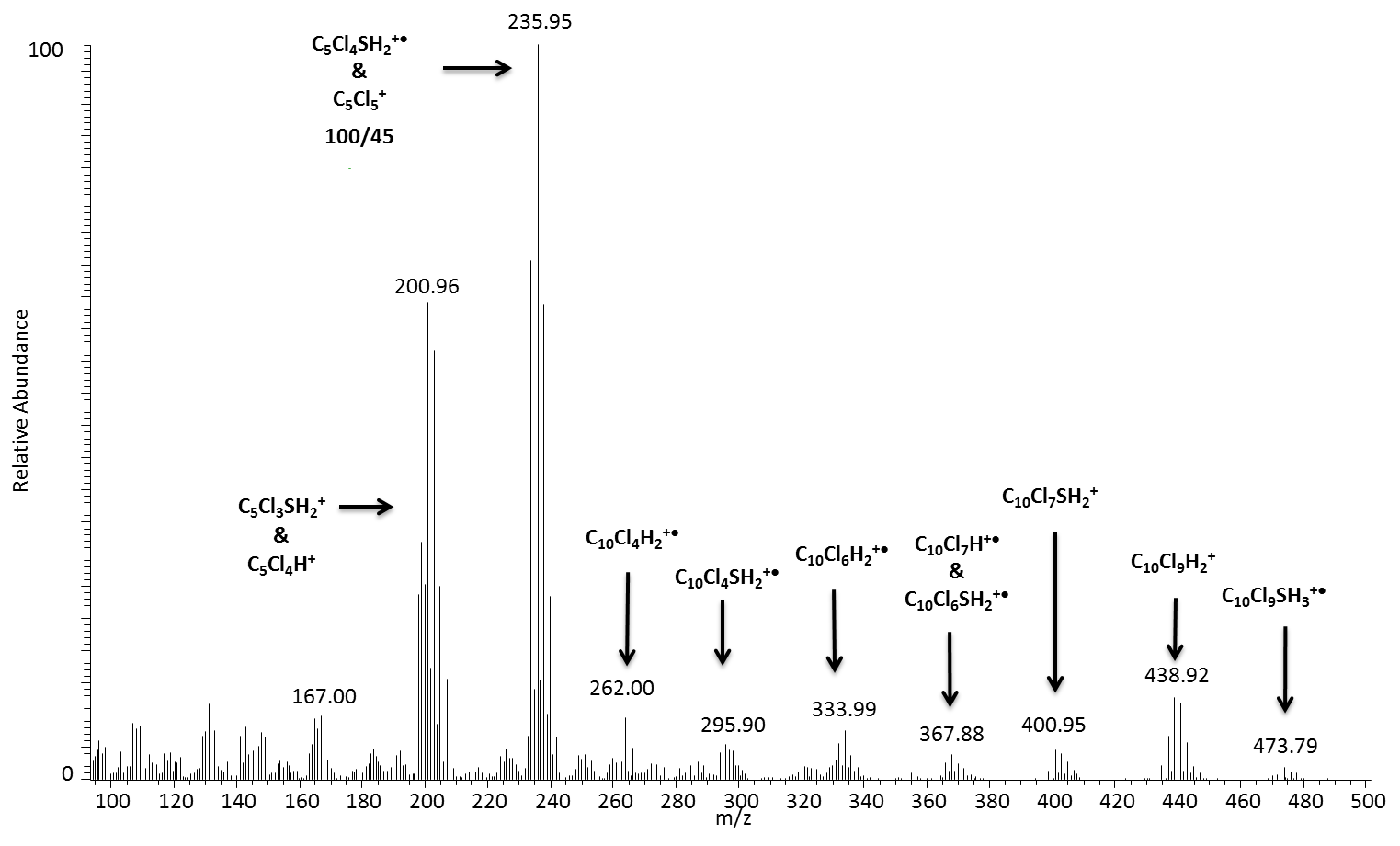


Figure S10: Mass spectrum of F3 from GC-EI-MS (positive mode) analysis and proposed in-source fragment ions assignment.

*
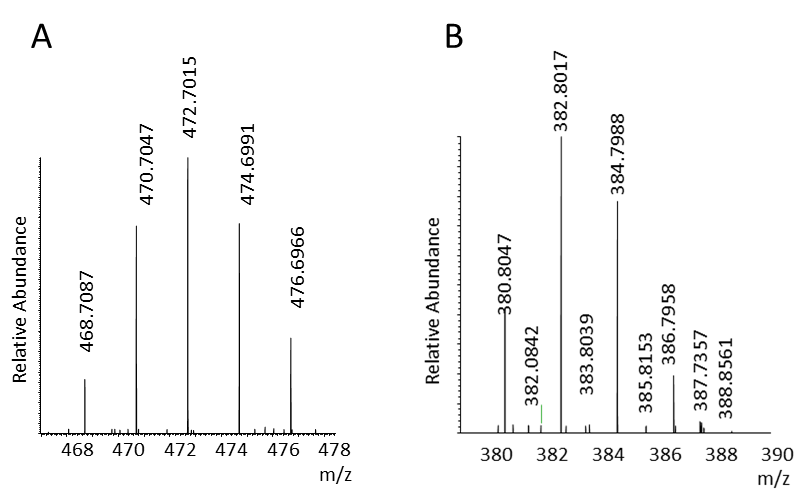
*

Figure S11: LC-HR mass spectrum of F2/F3.


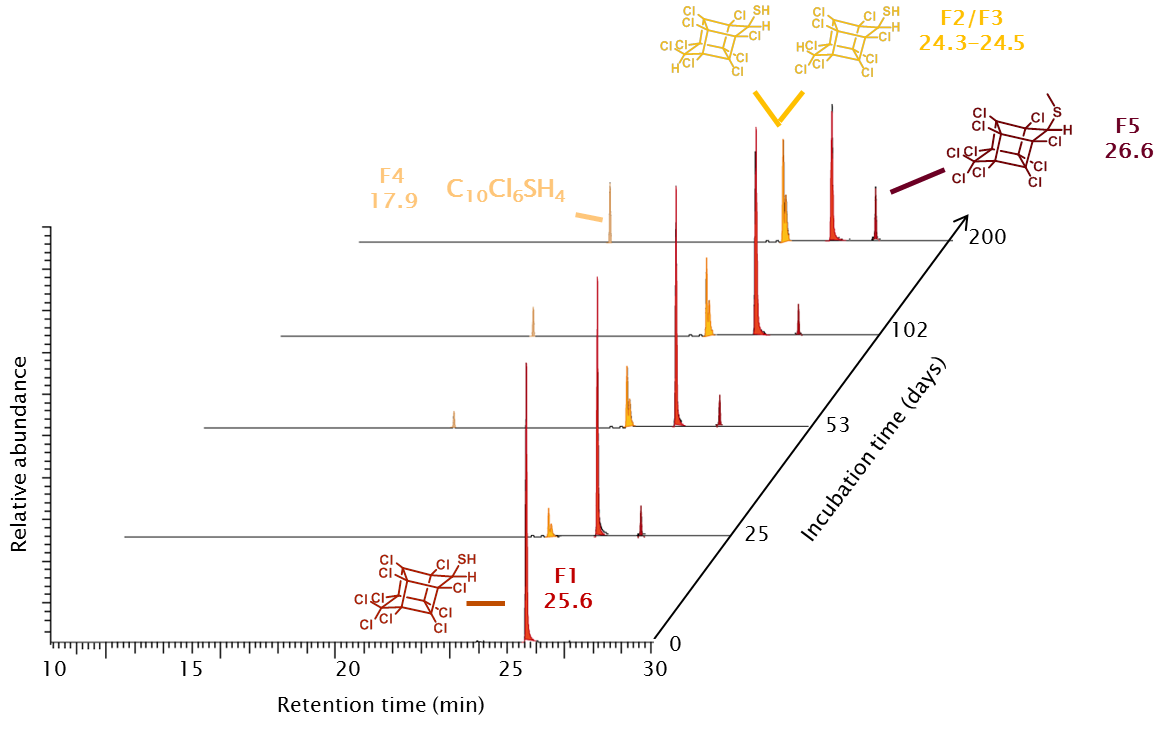


Figure S12: GC-MS monitoring of chlordecthiol transformation by *Desulfovibrio* sp.86 in RA conditions, MMD medium and N_2_/H_2_ (98/2, V/V) atmosphere in a glove box.


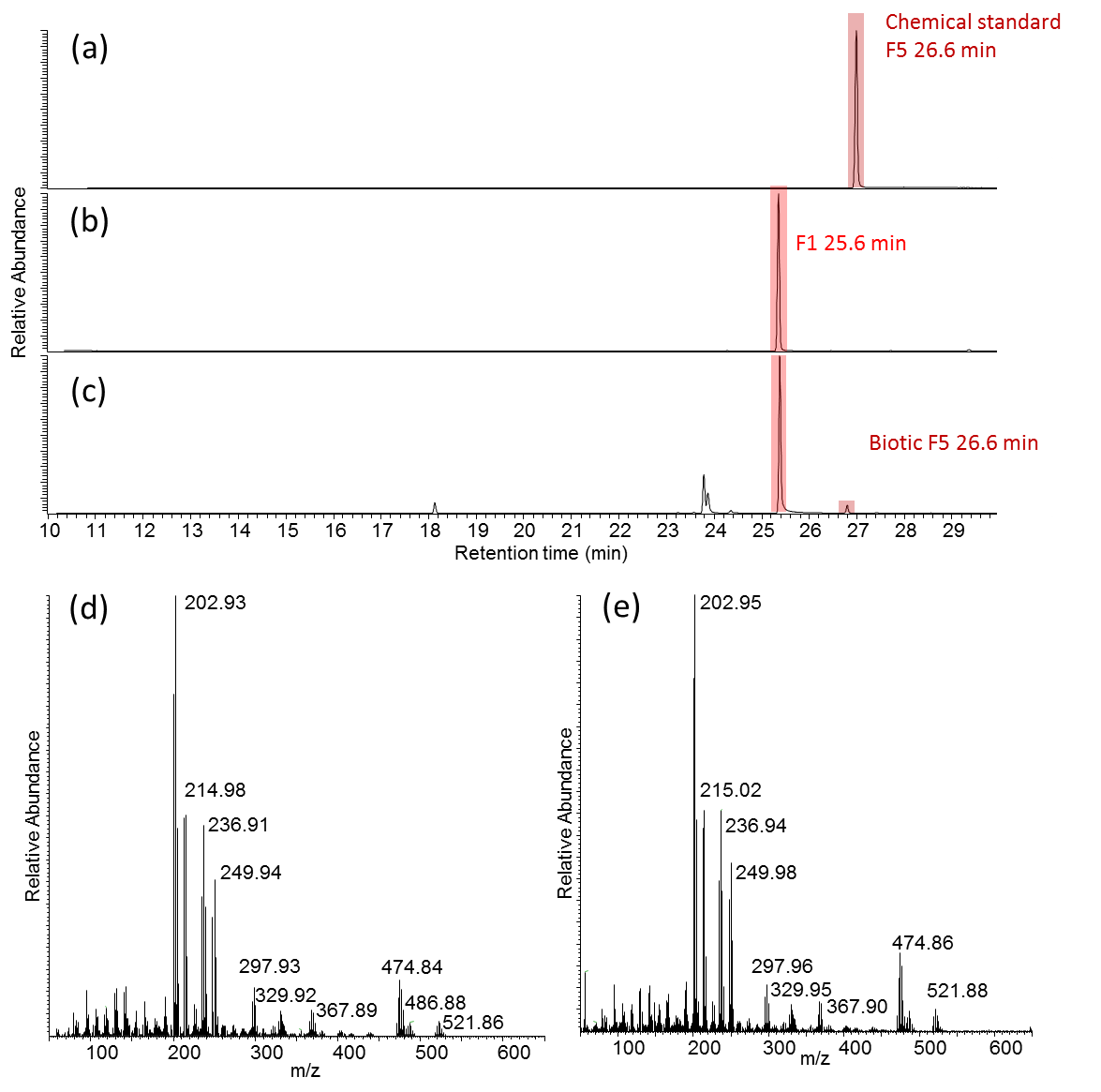


Figure S13: GC-MS analytical validation of F5 standard. (a) GC-MS chromatogram (full scan acquisition) of the chemical standard F5. (b) GC-MS chromatogram (full scan acquisition) of chlordecone. (c) GC-MS chromatogram (full scan acquisition) of a *Desulfovibrio* sp.86 culture incubated in CA condition, using MMD medium supplemented with F1, after an incubation period of 25 days. (d) Mass spectrum of the chemical standard F5 from GC-EI-MS (positive mode) analysis. (e) Mass spectrum of the microbial F5 from GC-EI-MS (positive mode) analysis.


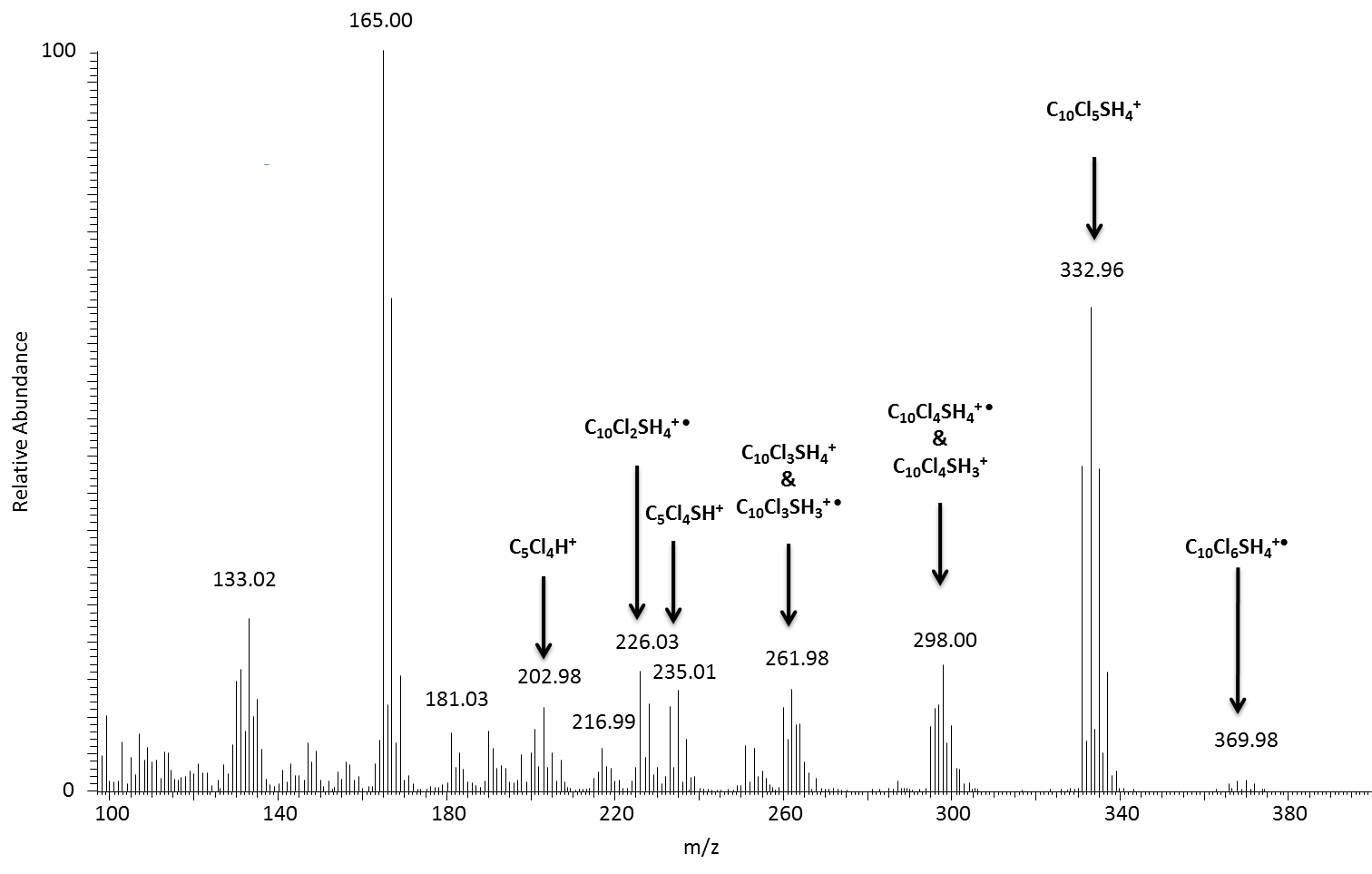


Figure S14: Mass spectrum of F4 from GC-EI-MS (positive mode) analysis and proposed in-source fragment ions assignment.


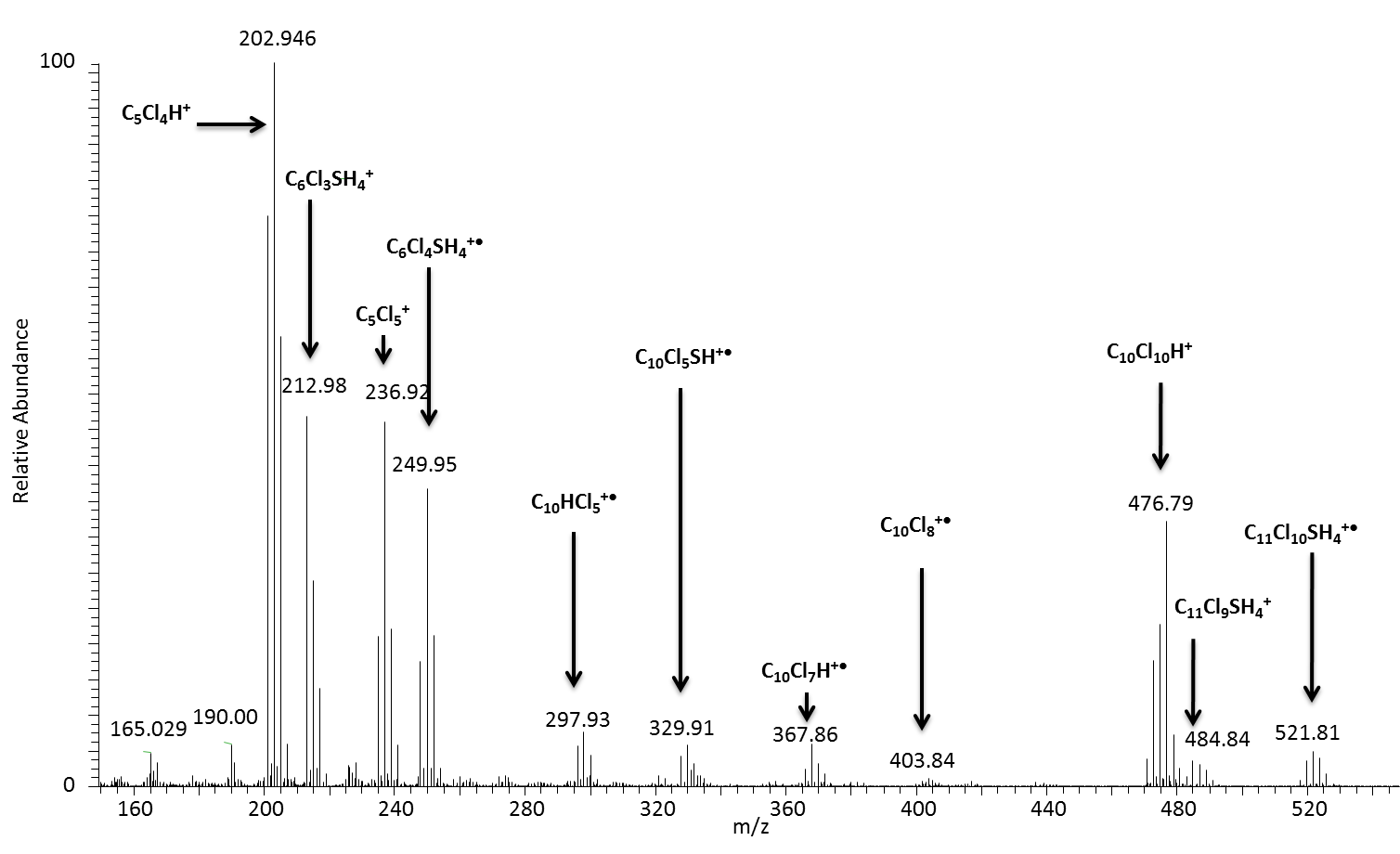


Figure S15: Mass spectrum of F5 from GC-EI-MS (positive mode) analysis and proposed in-source fragment ions assignment.

*
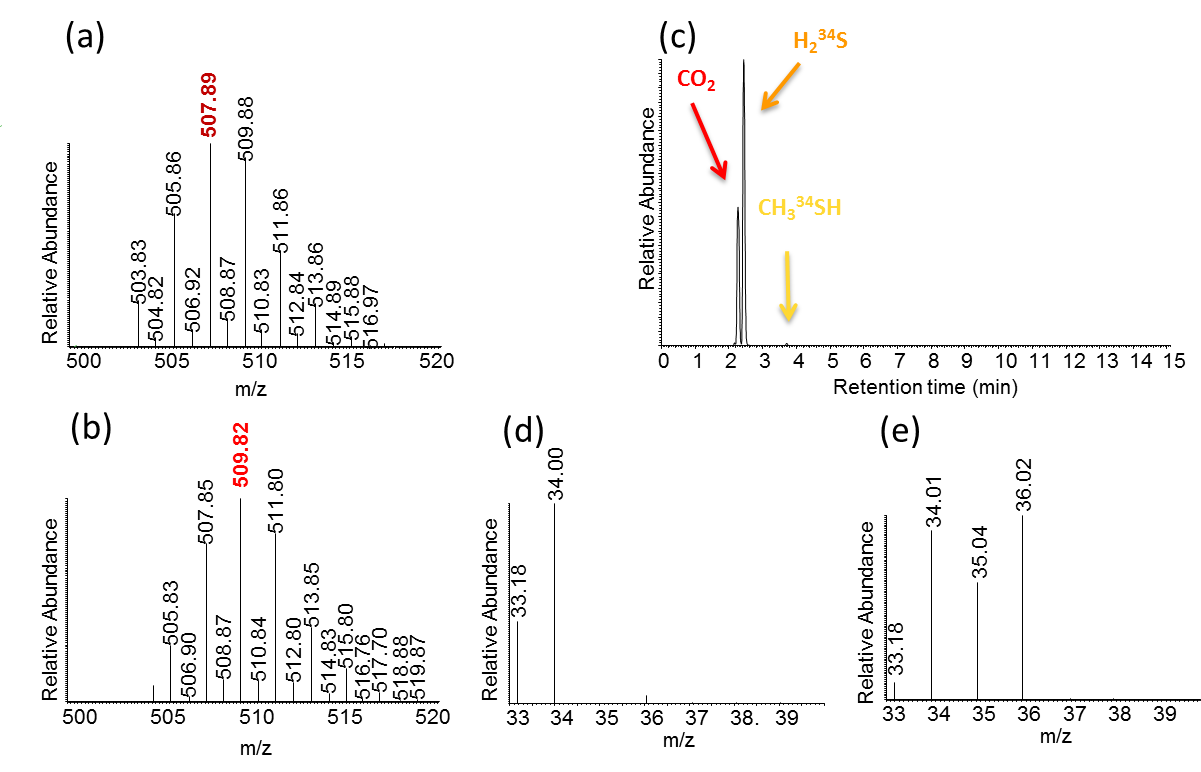
*

Figure S16: Comparison between cultures of *Desulfovibrio* sp.86, supplemented with chlordecone (40 mg/L), in MMD medium containing sulfate (7 mM) or ^34^S-labelled sulfate (containing 90% of ^34^S), in CA conditions. (a) Mass spectrum of F1 from GC-EI-MS (positive mode) generated by *Desulfovibrio* sp.86, supplemented with chlordecone (40 mg/L), in MMD medium containing sulfate (7 mM). (b) Mass spectrum of ^34^S enriched F1 from GC-EI-MS (positive mode) generated by *Desulfovibrio* sp.86, supplemented with chlordecone (40 mg/L), in MMD medium containing ^34^S-labelled sulfate. (c) Chromatogram of the ^34^S-labelled sulfate culture of *Desulfovibrio* sp-86 gas phase culture in HS-GC-MS (corresponding to the volume of gas present in the sealed vial). (d) Mass spectrum of synthetic H_2_S from GC-EI-MS (positive mode). (e) Mass spectrum of ^34^S enriched H_2_S from GC-EI-MS (positive mode), coming from the ^34^S-labelled sulfate culture of *Desulfovibrio* sp-86.


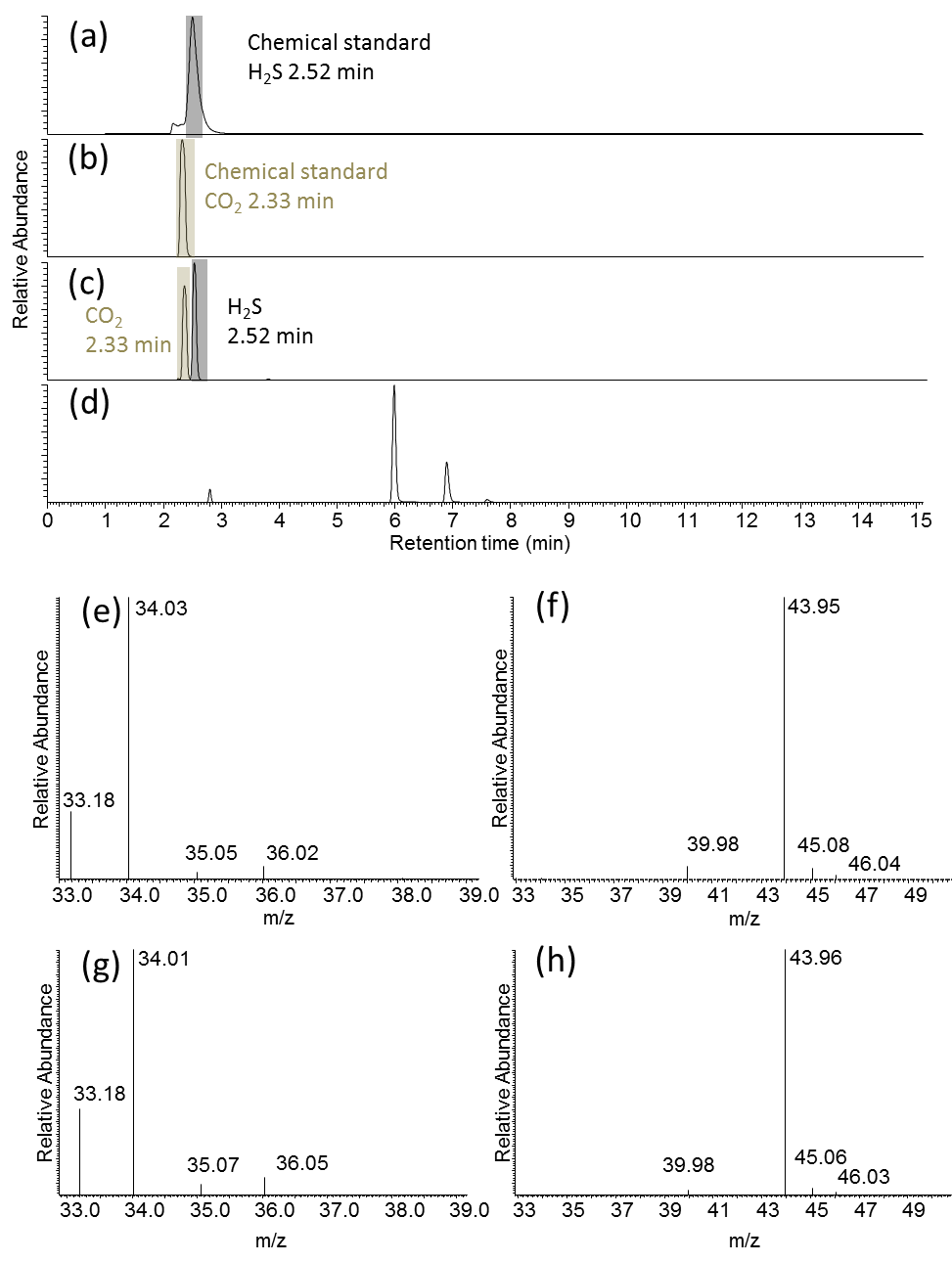


Figure S17: HS-GC-MS detection of H_2_S in specific *Desulfovibrio* sp.86 cultures . (a) GC-MS chromatogram of the chemical standard H_2_S, produced from the hydrolysis of P_4_S_10_ (extract ion chromatogram). (b) GC-MS chromatogram of the chemical standard CO_2_, produced from the reaction between K_2_CO_3_ and HCl (extract ion chromatogram). (c) HS-GC-MS chromatogram of a *Desulfovibrio* sp.86 culture incubated in CA condition, using MMD medium after an incubation period of 2 months (full scan acquisition). (d) HS-GC-MS chromatogram of a *Desulfovibrio* sp.86 culture incubated in RA condition, using MMD medium after an incubation period of 2 months (full scan acquisition). (e, f) Mass spectra of the chemical standards H_2_S and CO_2_ respectively from GC-EI-MS (positive mode) analysis. (g, h) Mass spectra of the microbial H_2_S and CO_2_ respectively from GC-EI-MS (positive mode) analysis.


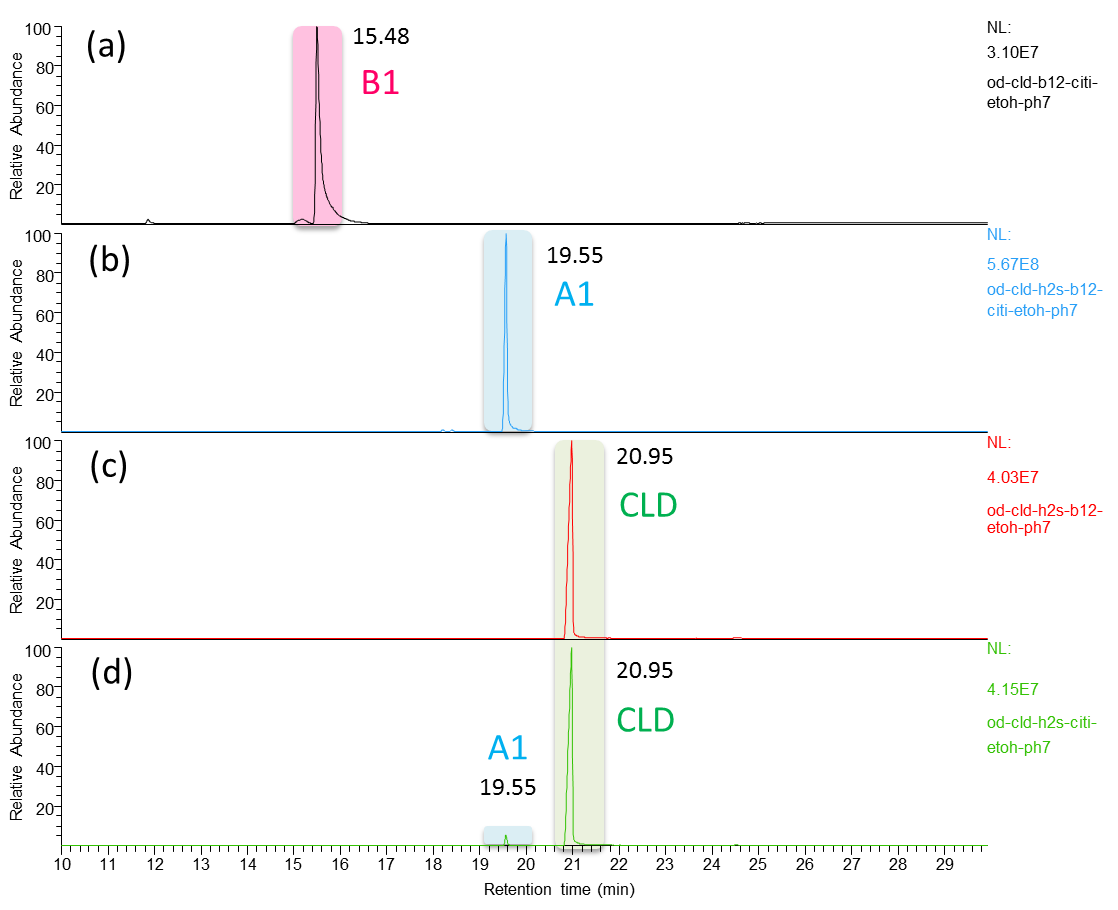


Figure S18: GC-MS chromatograms obtained after 80 mins of CLD chemical transformation according to reaction conditions: (a) chlordecone (1 equivalent (eq)), vitamin B_12_ (0.3 eq), CiTi (III) (32 eq) under N_2_ atmosphere, (b) chlordecone (1 eq), vitamin B_12_ (0.3 eq), CiTi (III) (32 eq) under H_2_S atmosphere, (c) chlordecone (1 eq), vitamin B_12_ (0.3 eq), under H_2_S atmosphere, (d) chlordecone (1eq), CiTi (III) (32 eq) under H_2_S atmosphere.

# S6 Supplementary NMR Spectra


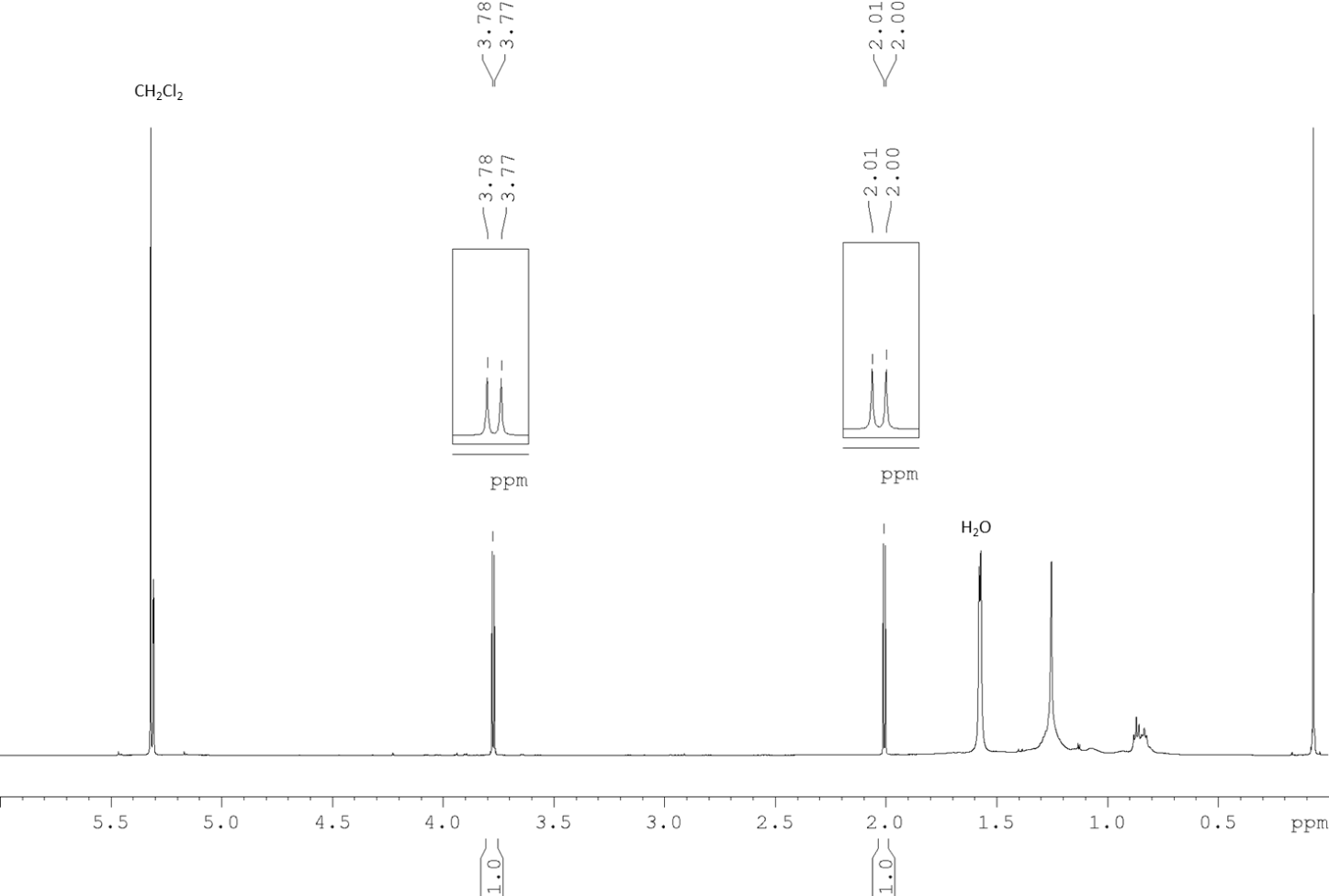


Figure S19: ^1^H-NMR spectrum of chlordecthiol in CD_2_Cl_2_ (600 MHz). *δ* 3.78 (d, 1 H, *J* = 5.5 Hz, C5-*H*(SH)), 2.01 (d, 1 H, *J* = 5.5 Hz, C5-H(S*H*)). [M-H]- calcd. for C_10_Cl_10_SH_2_, 502.6679; found, 502.6695. TLC: Rf (cyclohexane : acetone 10/1) = 0.23.


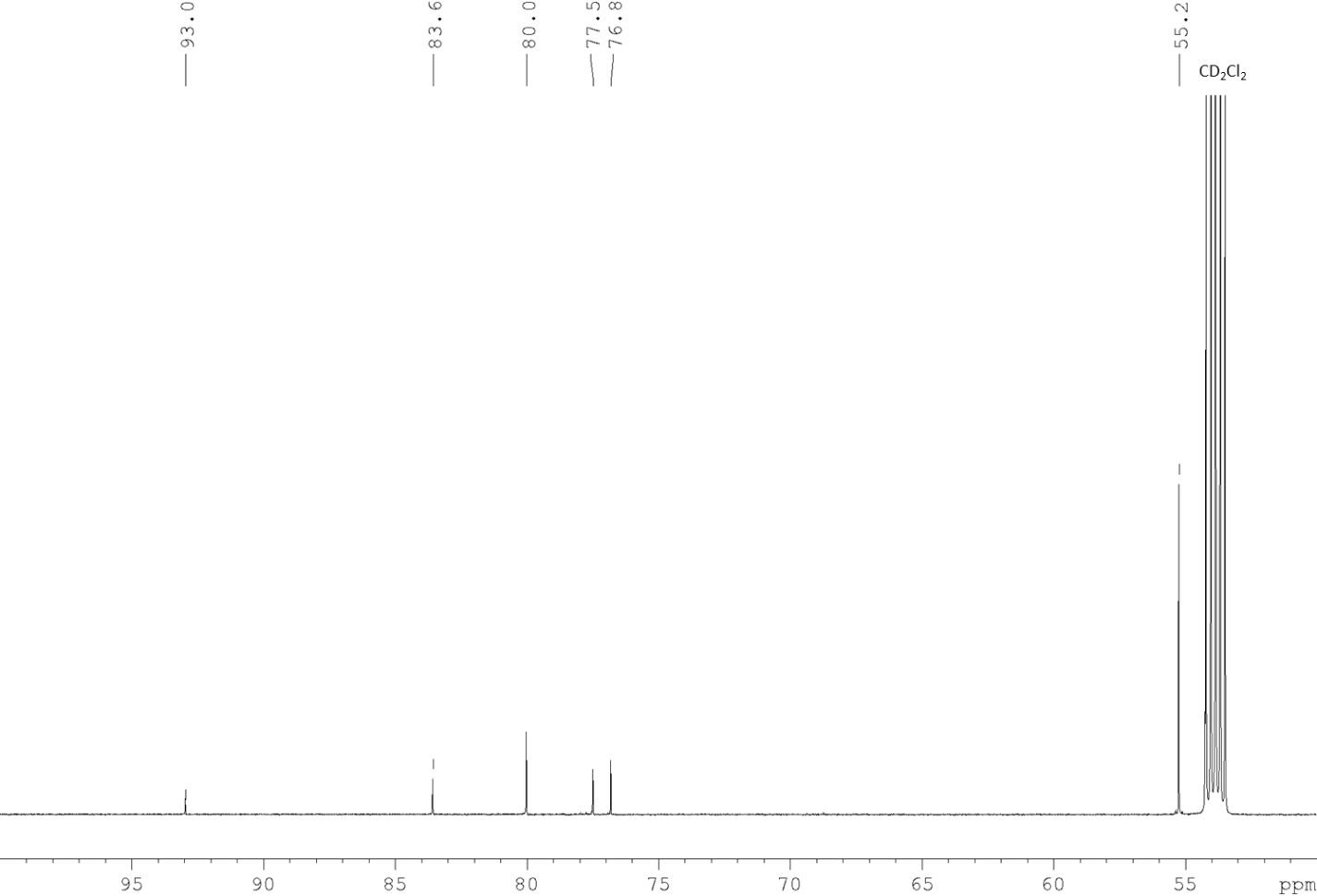


Figure S20: ^13^C-NMR spectrum of chlordecthiol in CD_2_Cl_2_ (150 MHz). *δ* 93.0 (C10), 83.6 (C1-C9), 80.0 (C4-C6), 77.5 (C2-C3), 76.8 (C7-C8), 55.2 (C5).


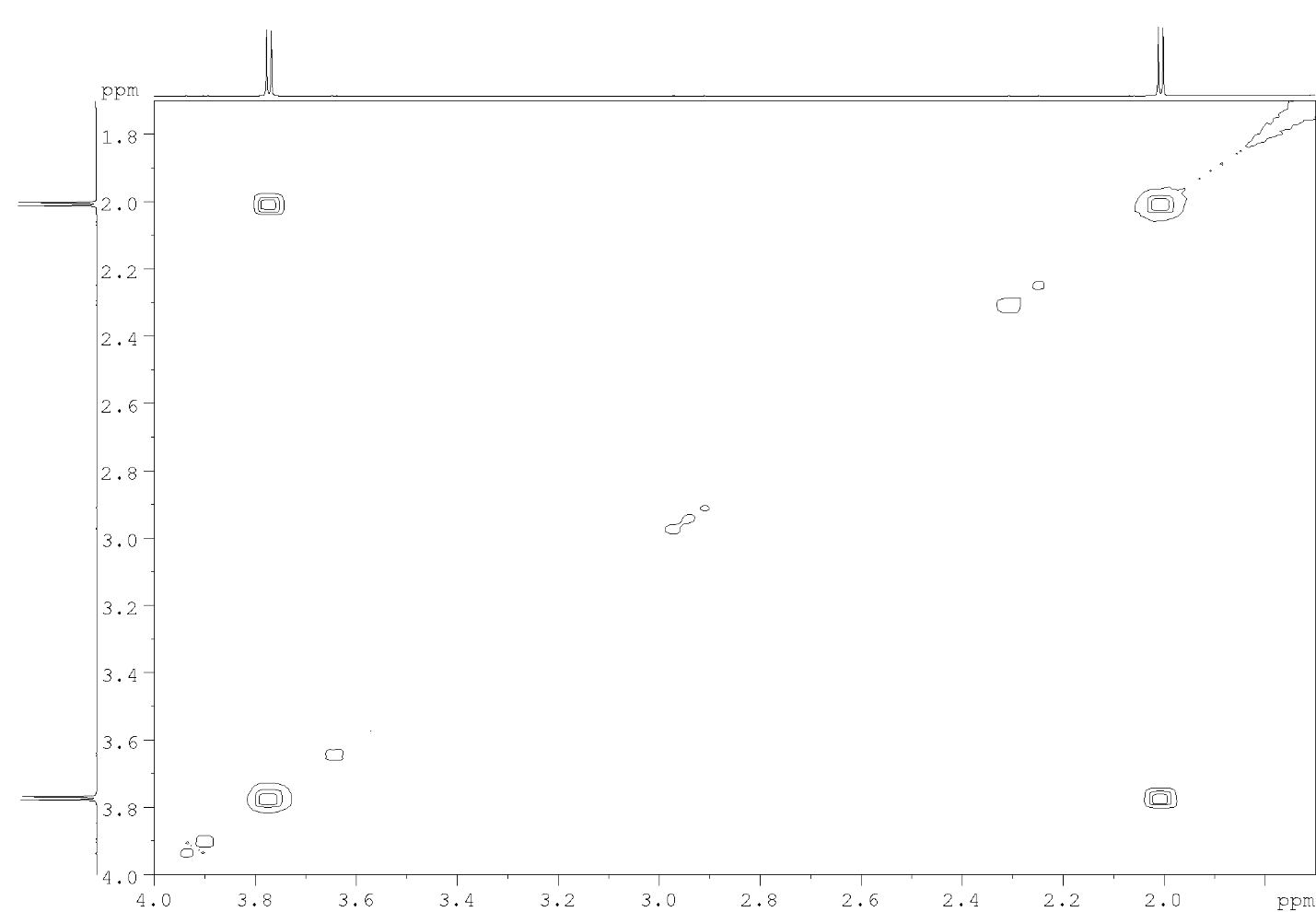


Figure S21: COSY spectrum of chlordecthiol in CD_2_Cl_2_ (600 MHz).


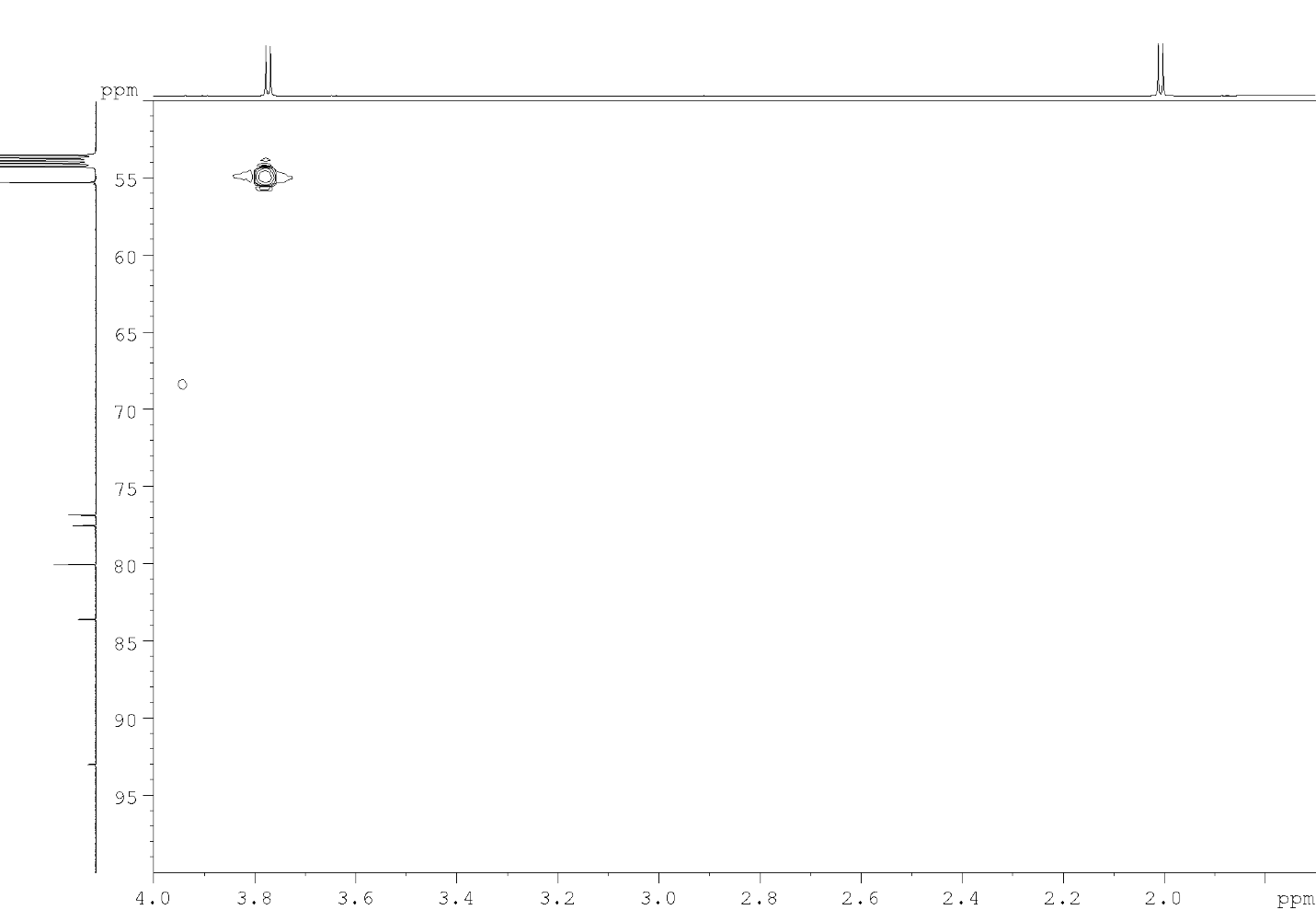


Figure S22: HSQC spectrum of chlordecthiol in CD_2_Cl_2_.


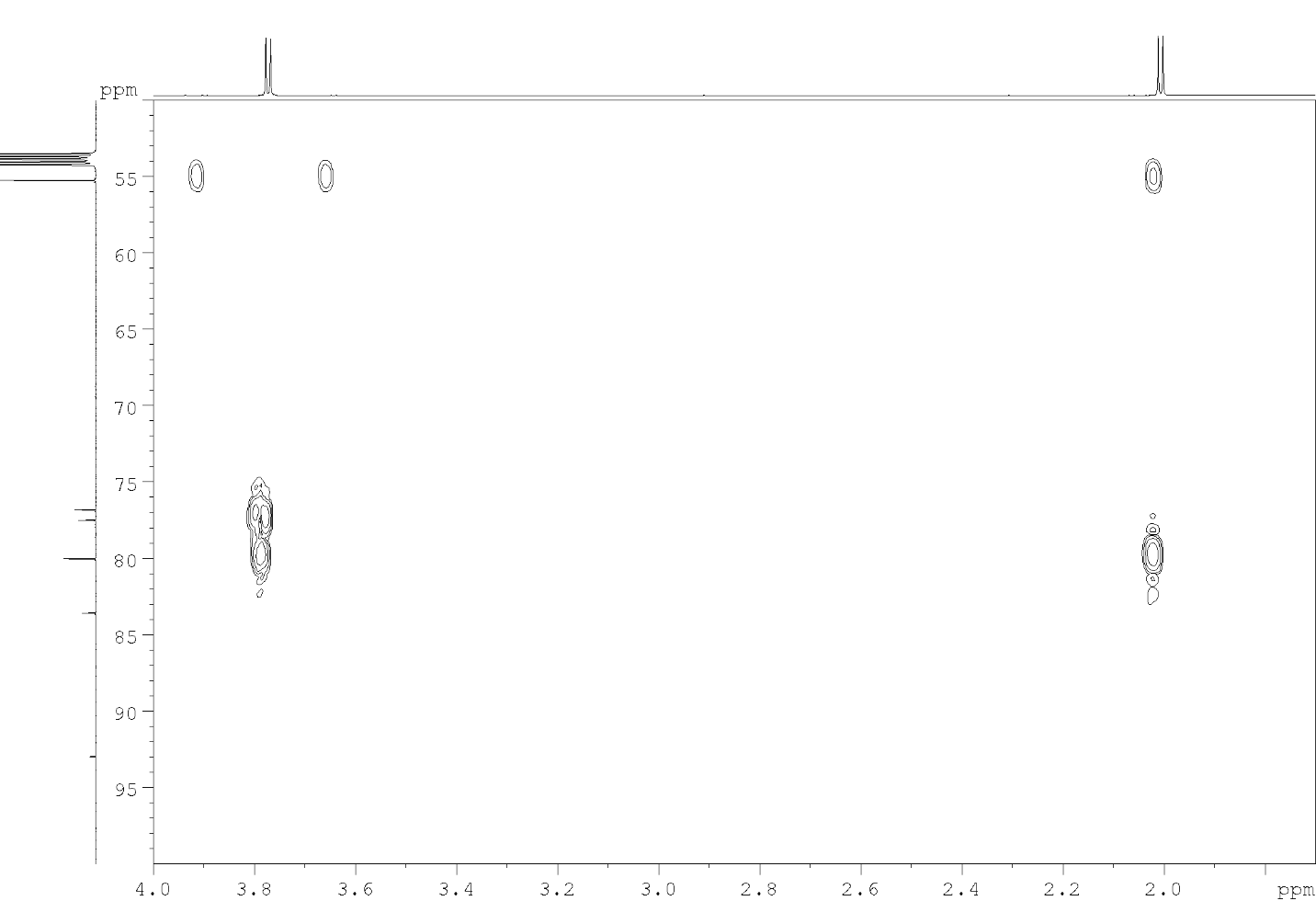


Figure S23: HMBC spectrum of chlordecthiol in CD_2_Cl_2_.


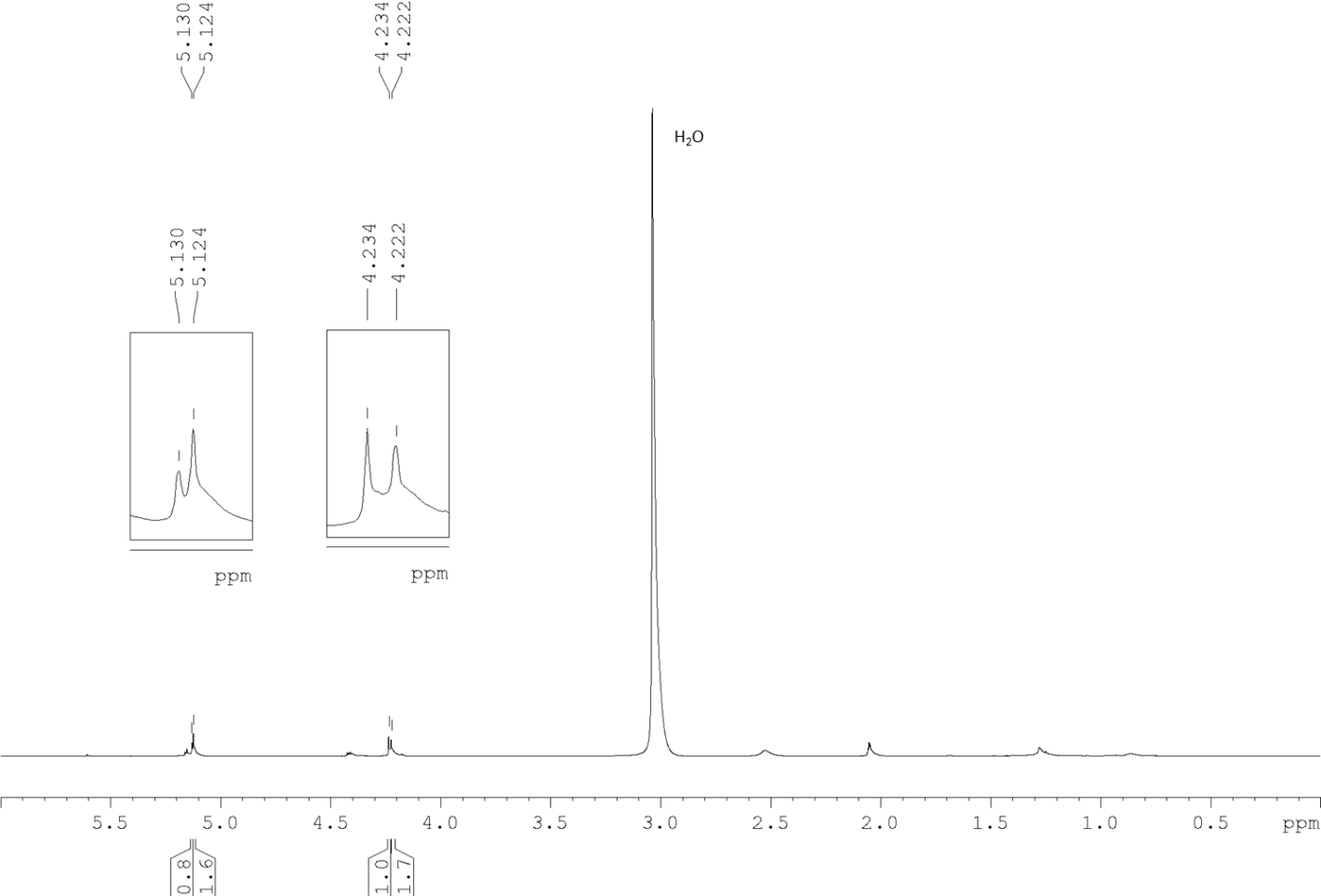


Figure S24: ^1^H-NMR spectrum of F2/F3 in CO(CD_3_)_2_ (600 MHz). *F(****2****)δ* 5.13 (s, 1 H, C10-*H*(Cl)), 4.23 (s, 1 H, C5-H(S*H*)). *F(****3****)δ* 5.12 (s, 1 H, C10-*H*(Cl)), 4.22 (s, 1 H, C5-H(S*H*)). [M-H]- calcd. for C_10_Cl_9_SH_3_, 468.7087; found, 468.7068. TLC: Rf (cyclohexane : acetone 10/1) = 0.23


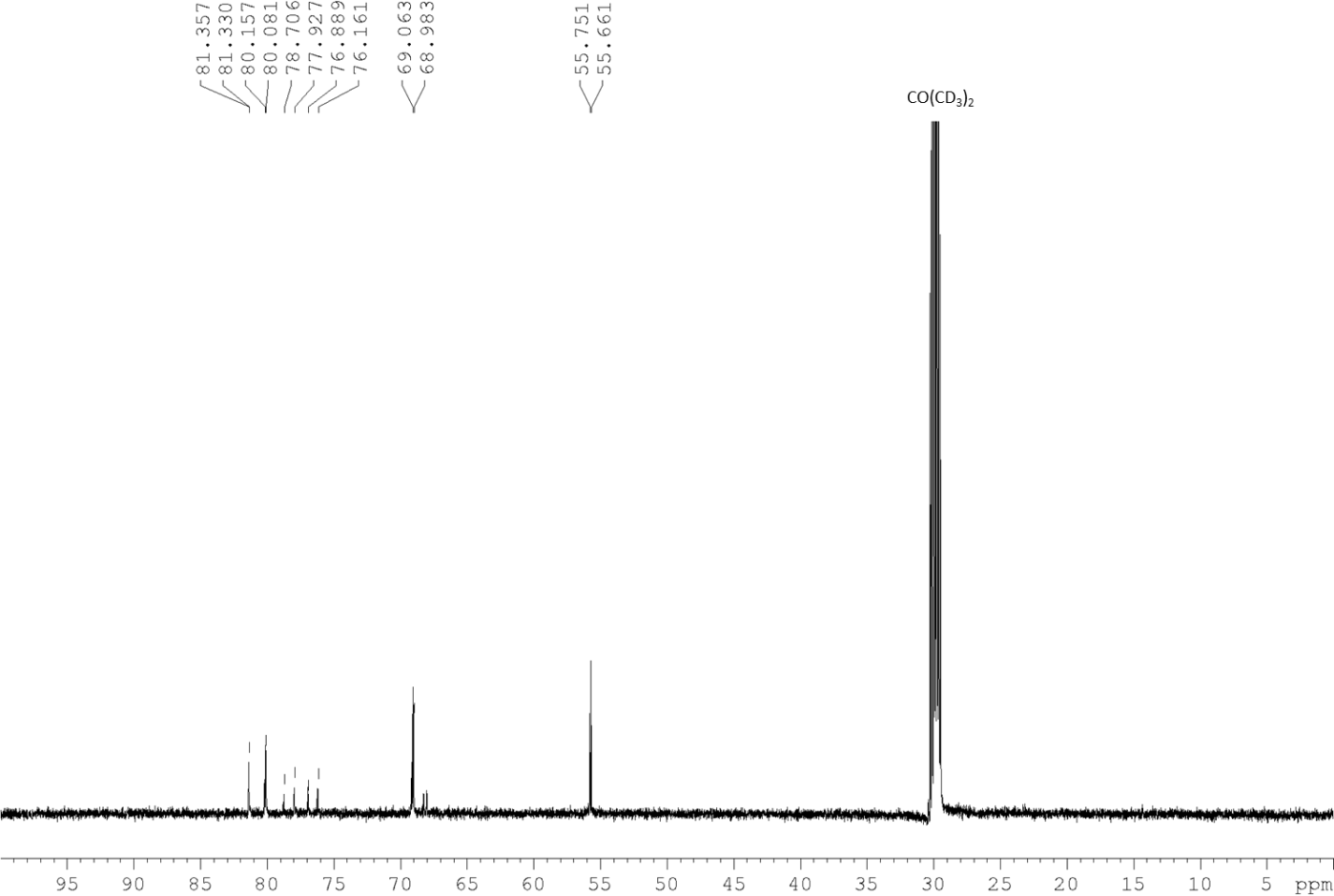


Figure S25: ^13^C-NMR spectrum of F2/F3 in CO(CD_3_)_2_ (150 MHz). *F(****2****)δ* 81.3 (C4-C6), 80.2 (C1-C9), 77.9 (C7-C8), 76.9 (C2-C3), 69.9 (C10), 55.8 (C5). *F(****3****)δ* 81.4 (C4-C6), 80.1 (C1-C9), 78.1 (C7-C8), 76.2 (C2-C3), 70.0 (C10), 55.7 (C5).


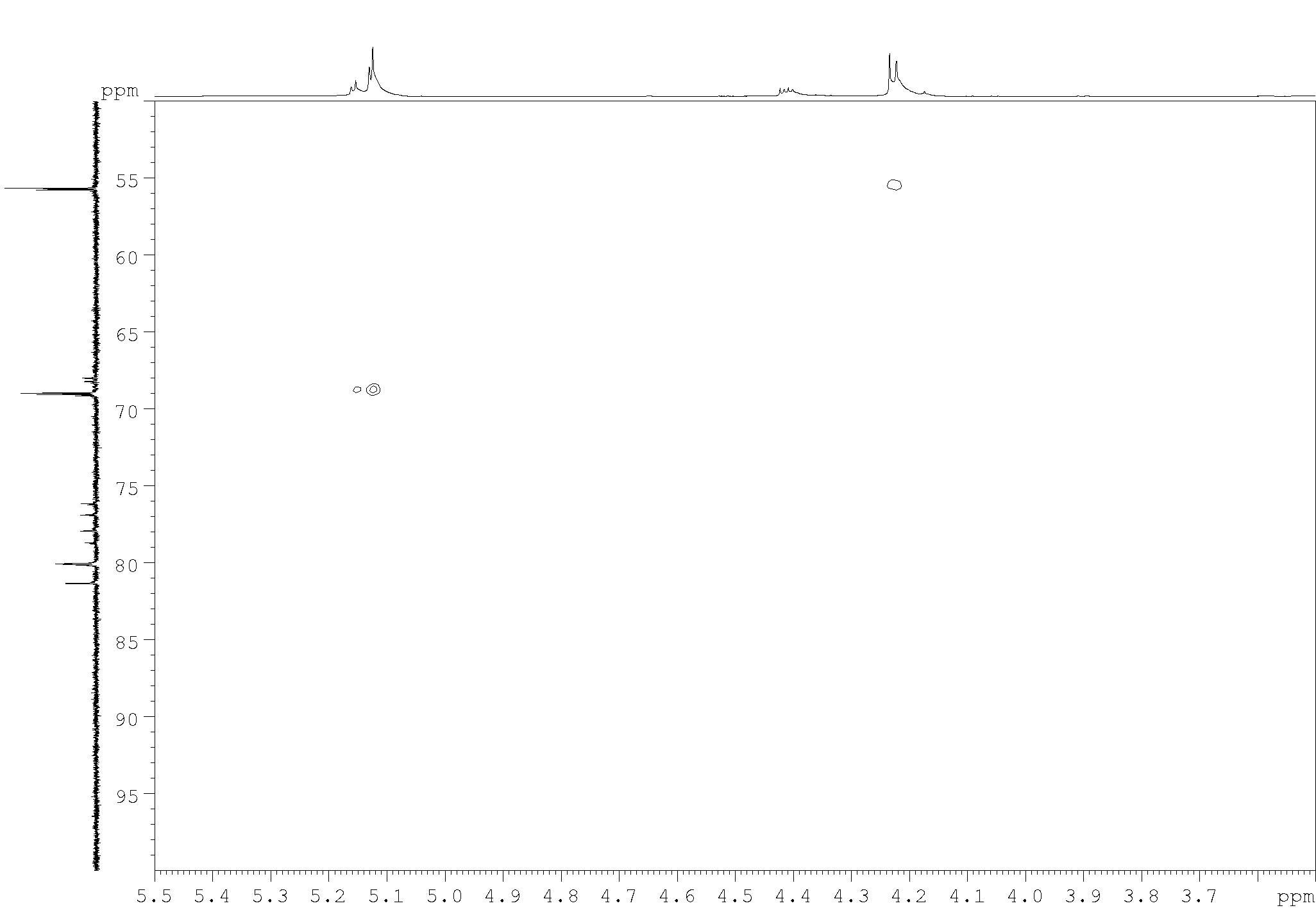


Figure S26: HSQC spectrum of F2/F3 in CO(CD_3_)_2_.


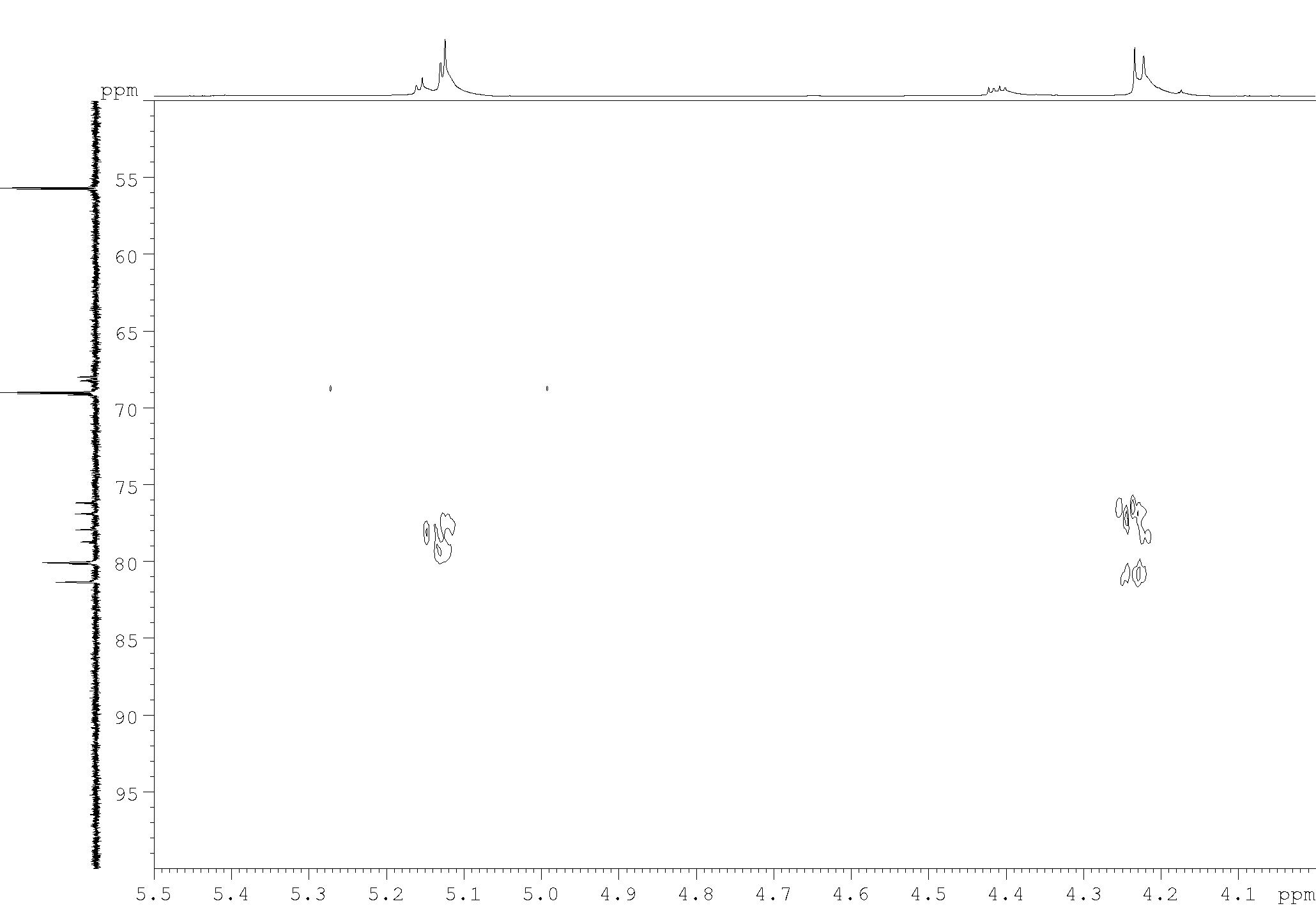


Figure S27: HMBC spectrum of F2/F3 in CO(CD_3_)_2_.


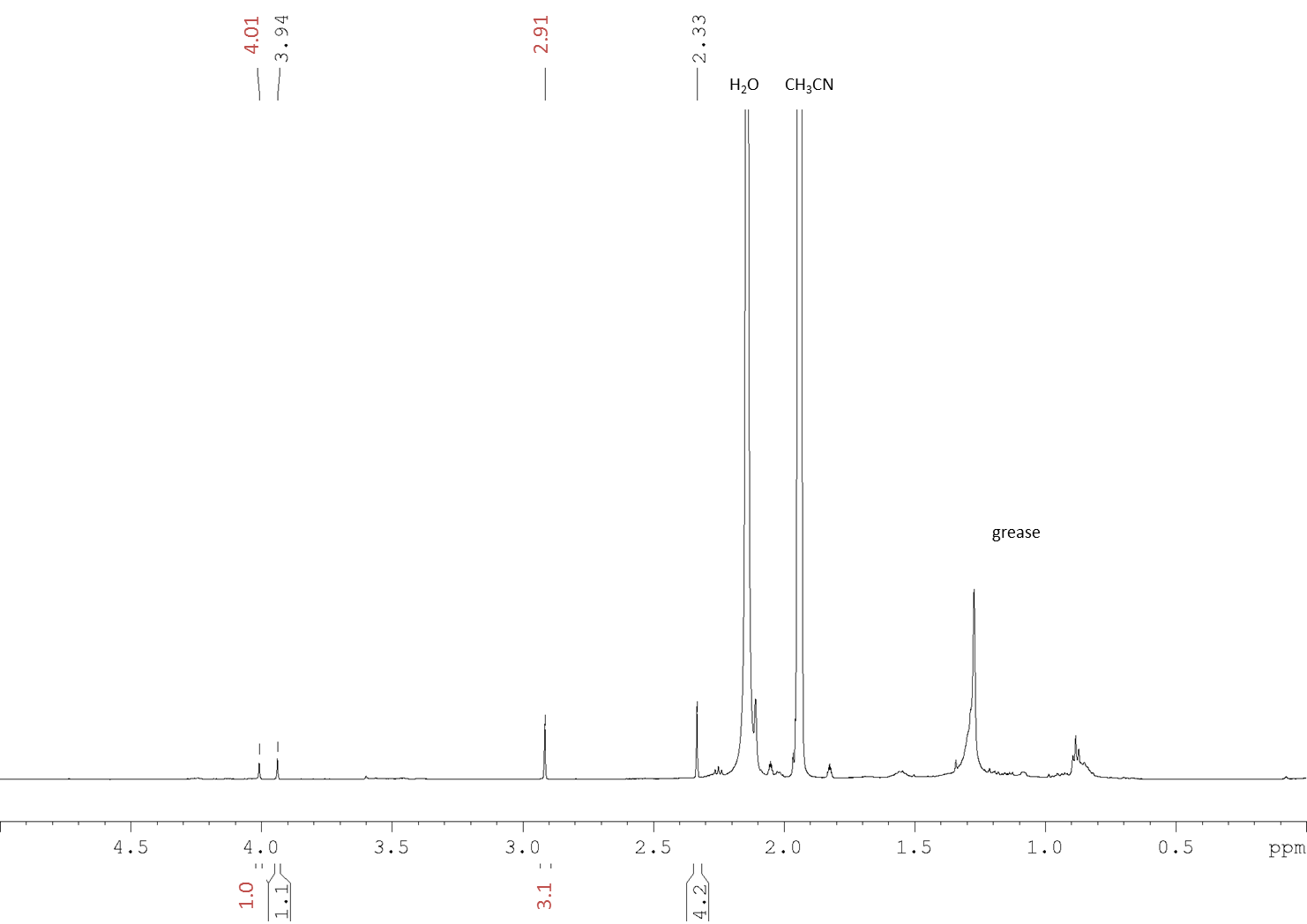


Figure S28: ^1^H-NMR spectrum of methyl chlordecsulfide in acetonitrile-d_3_ (600 MHz). *δ* 3.94 (s, 1 H, C5-*H*(SCH_3_)), 2.33 (s, 3 H, C5-H(SC*H_3_*)).. TLC: Rf (cyclohexane : acetone 10/1) = 0.69. Red attributions correspond to F5 oxidized form.


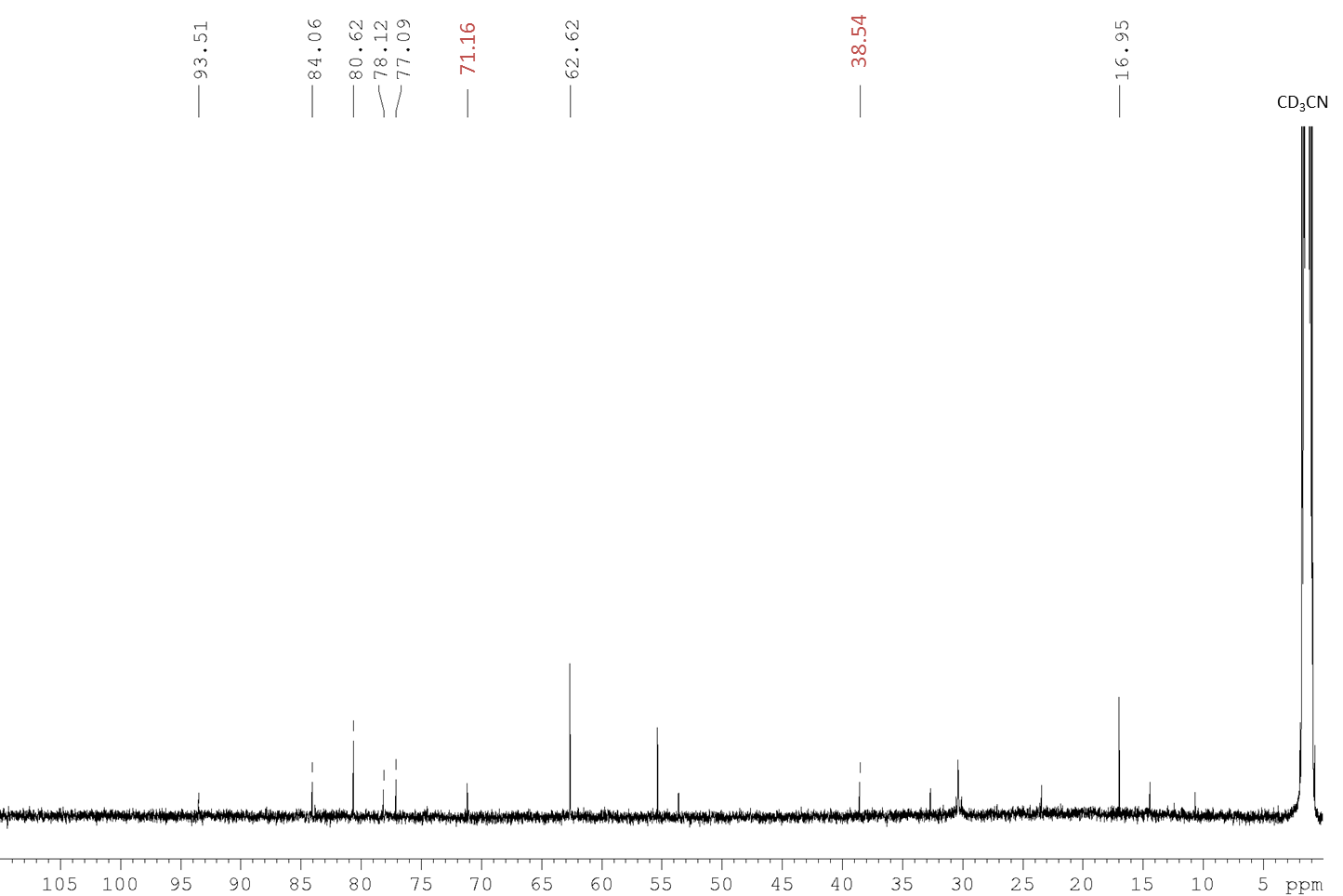


Figure S29: ^13^C-NMR spectrum of methyl chlordecsulfide in acetonitrile-d_3_ (150 MHz). *δ* 93.5 (C10), 84.1(C1-C9), 80.6 (C4-C6), 78.1(C2-C3), 77.1(C7-C8), 62.6 (C5), 17.0 (C11). Red attributions correspond to F5 oxidized form.


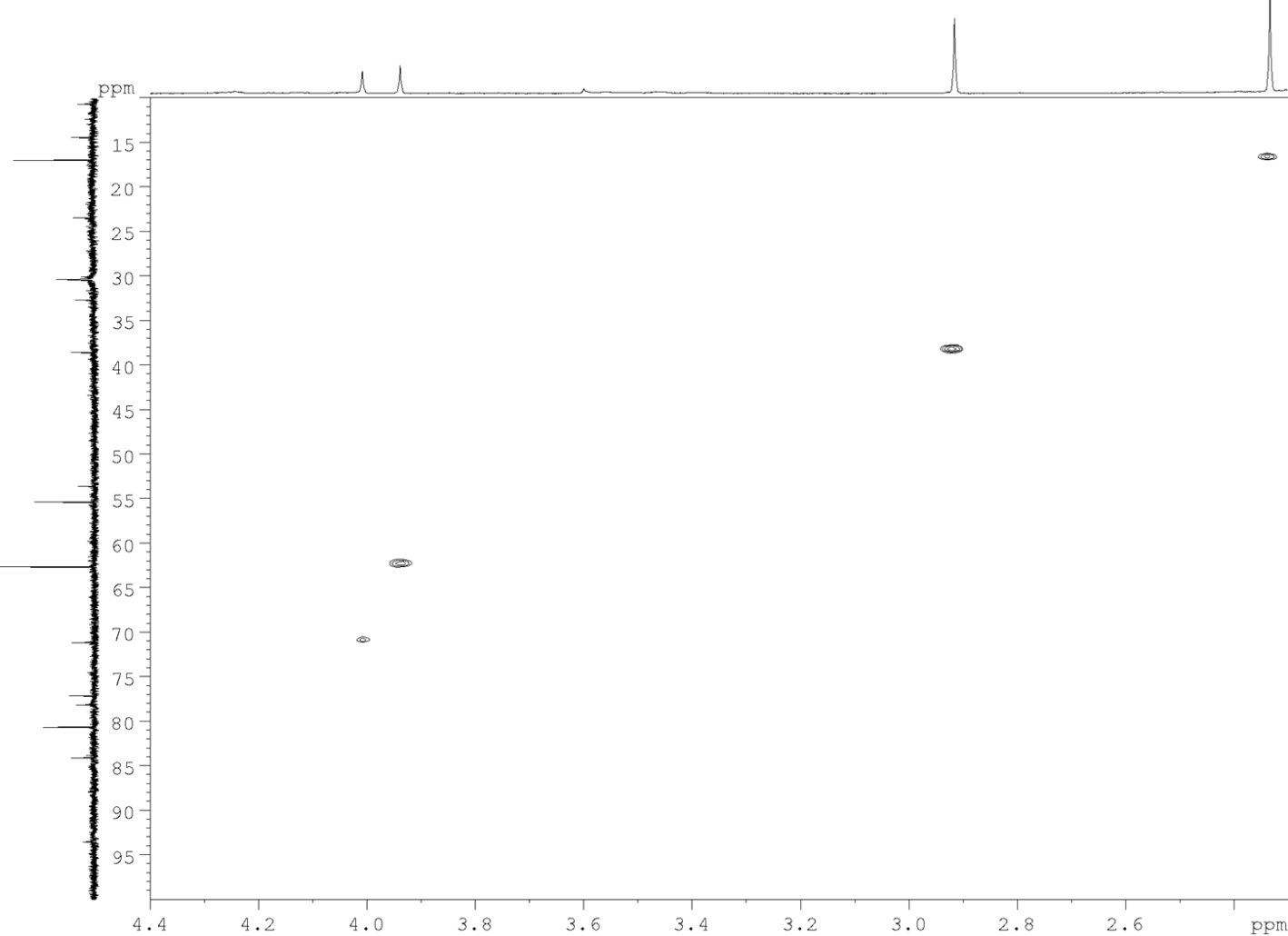


Figure S30: HSQC spectrum of methyl chlordecsulfide in acetonitrile-d_3_.


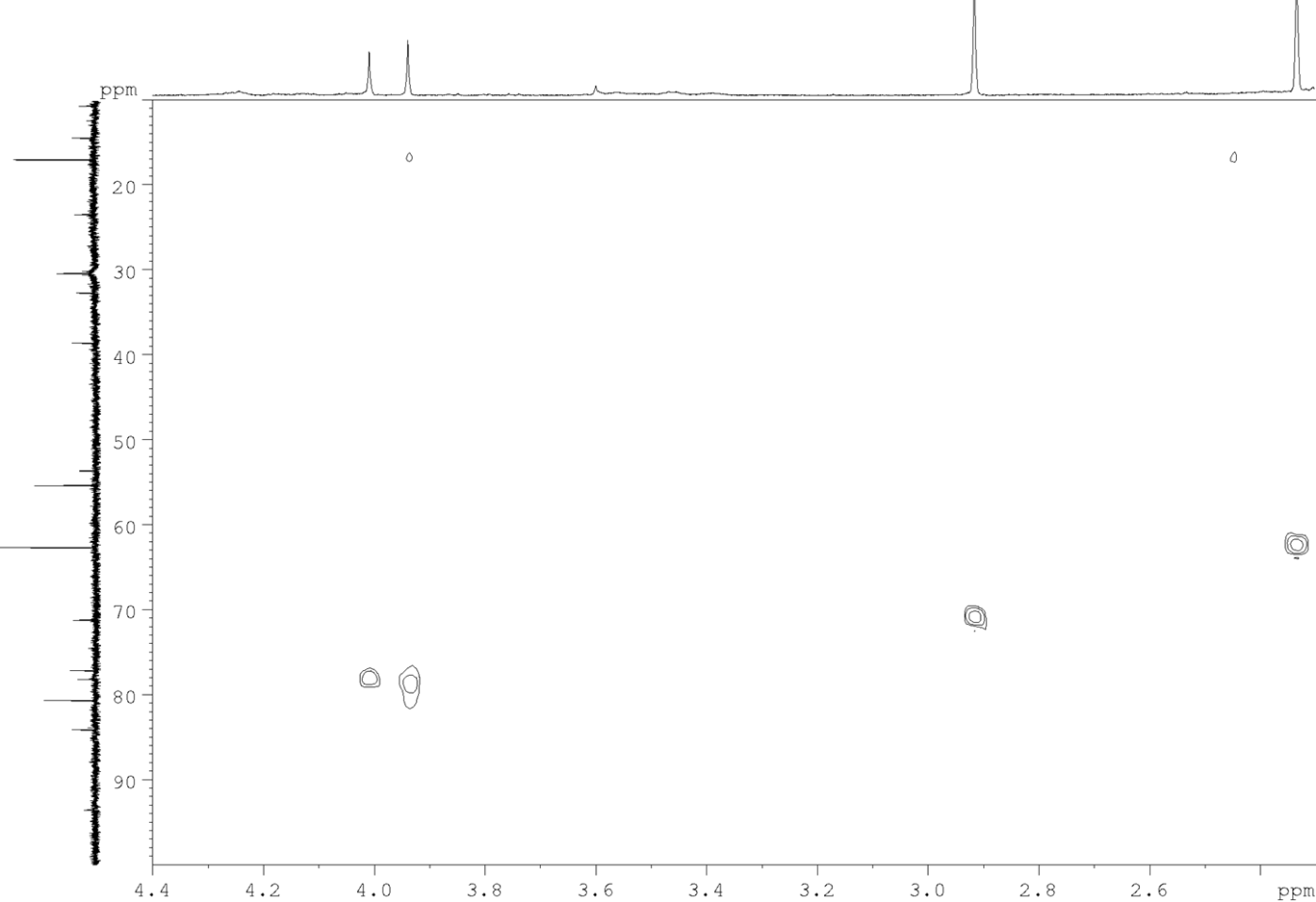


Figure S31: HMBC spectrum of methyl chlordecsulfide in acetonitrile-d_3_.

# S7 Supplementary References

1. Edgar, R. C. Search and clustering orders of magnitude faster than BLAST. *Bioinformatics* **26**, (19), 1, doi.org/10.1093/bioinformatics/btq461 (2010).

2. Kopylova, E., Noé, L., Touzet, H. SortMeRNA: fast and accurate filtering of ribosomal RNAs in metatranscriptomic data. *Bioinformatics,* **28**, (24), 6, doi.org/10.1093/bioinformatics/bts611 (2012).

3. Chaussonnerie, S., Ugarte, E., Saaidi, P-L., *et al.* Microbial Degradation of a Recalcitrant Pesticide: Chlordecone. *Front. Microbiol.* **7**, 2025, doi:10.3389/fmicb.2016.02025 (2016).

4. Ondov, B. D. *et al*. Mash: fast genome and metagenome distance estimation using MinHash. *Genome Biol.* **17**, 132, doi:10.1186/s13059-016-0997-x (2016).

5. Vallenet, D. *et al.* MicroScope: an integrated platform for the annotation and exploration of microbial gene functions through genomic, pangenomic and metabolic comparative analysis. *Nucleic Acids Res.* **48**, D579-D589, doi:10.1093/nar/gkz926 (2020).

6. Letunic, I., Bork, P. Interactive Tree Of Life (iTOL) v4: recent updates and new developments. *Nucleic Acids Res.* **47***,*  W256-W259, doi:10.1093/nar/gkz239 (2019).
